# Prevalent and persistent new-onset autoantibodies in mild to severe COVID-19

**DOI:** 10.1038/s41467-024-53356-5

**Published:** 2024-10-17

**Authors:** August F. Jernbom, Lovisa Skoglund, Elisa Pin, Ronald Sjöberg, Hanna Tegel, Sophia Hober, Elham Rostami, Annica Rasmusson, Janet L. Cunningham, Sebastian Havervall, Charlotte Thålin, Anna Månberg, Peter Nilsson

**Affiliations:** 1 Division of Affinity Proteomics, Department of Protein Science, SciLifeLab, KTH Royal Institute of Technology, Stockholm, Sweden; 2 Division of Protein Technology, Department of Protein Science, KTH Royal Institute of Technology, Stockholm, Sweden; 3 Section of Neurosurgery, Department of Medical Sciences, Uppsala University Hospital, Uppsala, Sweden; 4 Department of Neuroscience, Karolinska Institutet, Stockholm, Sweden; 5 Department of Medical Sciences, Psychiatry, Uppsala University, Uppsala, Sweden; 6 Department of Clinical Sciences, Karolinska Institutet, Danderyd Hospital, Stockholm, Sweden

**Keywords:** Proteomics, Viral infection, Autoimmunity, Assay systems

## Abstract

Autoantibodies have been shown to be implied in COVID-19 but the emerging autoantibody repertoire remains largely unexplored. We investigated the new-onset autoantibody repertoire in 525 healthcare workers and hospitalized COVID-19 patients at five time points over a 16-month period in 2020 and 2021 using proteome-wide and targeted protein and peptide arrays. Our results show that prevalent new-onset autoantibodies against a wide range of antigens emerged following SARS-CoV-2 infection in relation to pre-infectious baseline samples and remained elevated for at least 12 months. We found an increased prevalence of new-onset autoantibodies after severe COVID-19 and demonstrated associations between distinct new-onset autoantibodies and neuropsychiatric symptoms post-COVID-19. Using epitope mapping, we determined the main epitopes of selected new-onset autoantibodies, validated them in independent cohorts of neuro-COVID and pre-pandemic healthy controls, and identified sequence similarities suggestive of molecular mimicry between main epitopes and the conserved fusion peptide of the SARS-CoV-2 Spike glycoprotein. Our work describes the complexity and dynamics of the autoantibody repertoire emerging with COVID-19 and supports the need for continued analysis of the new-onset autoantibody repertoire to elucidate the mechanisms of the post-COVID-19 condition.

## Abstract (web summary)

Autoantibody immune responses could contribute to acute and post-COVID-19 symptoms. Here, the authors describe longitudinal autoantibody responses in a cohort of healthcare workers and hospitalised COVID-19 patients from Sweden.

## Introduction

In SARS-CoV-2 infection^1^ and other pulmonary viral infections^2^, preexisting anti-type I interferon autoantibodies have been detected in 5–20% of severe disease cases and may affect therapeutic strategies^3,4^. Several studies have detected the presence of established autoantibodies in COVID-19 patients^5–11^, although their clinical significance remains unclear. In addition, autoantibodies against a wide range of extracellular antigens have been detected in COVID-19, and a subset of these have been shown to antagonize cytokine signaling, be associated with increased viral loads and decreased T-cell and B-cell populations, and to increase disease severity in mouse models of COVID-19^12^. The total number of these autoantibodies in COVID-19 patients has been associated with disease severity^12^. However, autoantibody repertoires are notoriously individual-specific in both health and disease^13,14^, rendering associations to clinical symptoms and outcomes difficult without a longitudinal study design.

To this end, some studies of hospitalized patients with COVID-19 have investigated the development of autoantibodies against a selection of previously described^15^ or extracellular^16^ antigens. While these studies demonstrated the existence of new-onset autoantibodies in patients with severe COVID-19, they were constrained by the use of baseline samples collected after hospitalization and short follow-up times. This presented limitations in the evaluation of the persistence of new-onset autoantibodies and their association with the course of COVID-19.

In the months following COVID-19, an estimated 6% of individuals experience lasting symptoms such as cognitive dysfunction, fatigue, and shortness of breath^17,18^. These symptoms are collectively known as long COVID, post-acute sequelae of COVID-19, or post-COVID-19 condition and may occur after mild as well as severe acute disease^17^. There are many theories on the etiology of the post-COVID-19 condition, including viral persistence, persistent inflammation, and autoimmunity, including the emergence of new-onset autoantibodies^3,19–21^. In particular, neurological symptoms after SARS-CoV-2 infection, termed neuro-COVID, are suspected to stem from a dysregulated immune response with autoantibody involvement^5,22–25^, similar to other post-infectious neurological disorders^25,26^. However, a notable cross-sectional study of immune disruption in the post-COVID-19 condition could not identify any associations with autoantibodies^27^, indicating the need for a longitudinal study of this immune compartment.

During the COVID-19 pandemic, we developed a highly specific and sensitive multiplex bead array for SARS-CoV-2 serology^28^ which we have used to profile the serological response in several research projects, where the COMMUNITY (COVID-19 Immunity) study is a longstanding collaboration^29–31^. This ongoing longitudinal study enrolled 2149 healthcare workers (HCW) and 118 admitted COVID-19 patients at Danderyd Hospital, Sweden, between April and May 2020, with follow-up visits every four months.

In the present study, we extend the analysis within a subgroup of the COMMUNITY study cohort by profiling the dynamics of autoantibody repertoires across SARS-CoV-2 infection using proteome-wide and targeted in-house developed planar and bead arrays. The results reveal prevalent new-onset autoantibodies against a wide range of antigens which remain elevated for at least 12 months, are associated with neuropsychiatric symptoms post-COVID-19, and invoke molecular mimicry with the SARS-CoV-2 Spike protein fusion peptide.

## Results

**Fig. 1:**
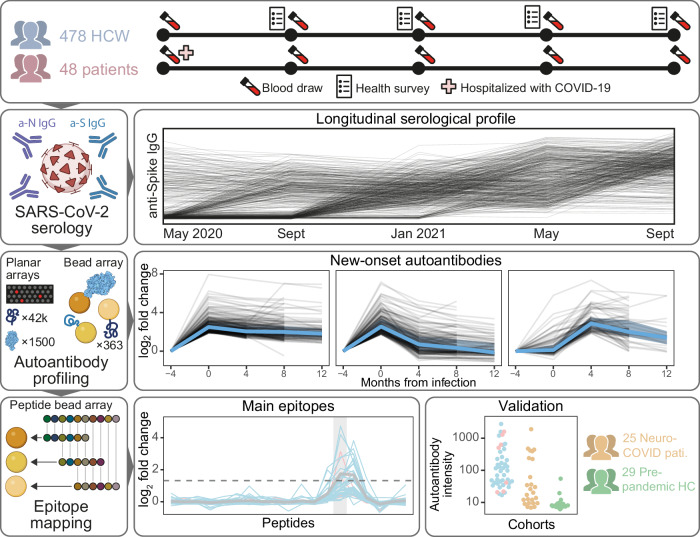
Study overview. Healthcare workers (HCW, *n* = 478) and patients (*n* = 48) with complete sample sets from May 2020 to Sept 2021 were selected for the study. Longitudinal serological profiles of anti-spike (S) and anti-nucleocapsid (N) immunoglobulin G (IgG) were obtained using our in-house SARS-CoV−2 serology assay. Proteome-wide planar and bead arrays were used to chart the IgG autoantibody repertoire across seroconversion for the identification of new-onset autoantibodies. Tiled peptide bead arrays were used to identify the main epitopes of selected new-onset autoantibodies and validate them in independent cohorts of Neuro-COVID patients (*n* = 25) and pre-pandemic healthy controls (HC, *n* = 29).

In this study, we have profiled the autoantibody repertoire of 478 HCW and 47 hospitalized COVID-19 patients and validated our results in 25 neuro-COVID patients and 29 pre-pandemic healthy controls (HCs) (Supplementary Table 1). An overview of the study is shown in Fig. 1. In summary, samples in the discovery cohorts were collected across 3–5 visits (mean 4.8) over 16 months, for a total of 2532 samples analyzed in the present study. In HCW, 20% (*n* = 96) were seropositive (had anti-SARS-CoV-2 immunoglobulin G (IgG)) at study inclusion in May 2020. Among the remaining 382 baseline seronegative HCW, 109 were seroconverted (first display of anti-SARS-CoV-2 IgG) in Sept 2020, 233 in Jan 2021, and 40 in May 2021. All HCW seroconverted before receiving their first SARS-CoV-2 vaccine dose. Among the patients, 85% (*n* = 40) were seropositive at the first sampling after admission (May 2020), and the remaining 15% (*n* = 7) had seroconverted at the first sampling after discharge (Sept 2020).

### Proteome-wide autoantibody profiling reveals diverse autoantibodies in COVID-19

To explore the proteome-wide autoantibody landscape emerging with COVID-19, we screened blood sample from 32 healthcare workers (HCW) with self-reported symptoms post-COVID-19, and 16 hospitalized COVID-19 patients, on our in-house developed planar array platforms. Plasma samples from the 32 HCW were divided into eight groups of four individuals each, defined by specific symptoms post-COVID-19 (Supplementary Table 2). Similarly, plasma samples from the 16 patients were divided into four groups of four individuals each, based on sex and comorbidities (Supplementary Table 2). Within each group, plasma samples were combined and analyzed on the arrays. HCW and patient groups were analyzed on the proteome-wide arrays containing 42,000 protein fragments, and HCW groups were, in addition, analyzed on the Secretome arrays containing 1522 full-length proteins.

In total, IgG binding was detected towards 215 protein fragments and 22 full-length proteins, with 14 to 36 reactive autoantibodies in each group. Autoantibody profiles were highly specific to each set of combined samples, with 6% (15 of 237) of autoantibodies being reactive in two groups and 3% (8 of 237) being reactive in three to six groups (Supplementary Fig. 1). The reactive antigens were selected for further investigation in the full HCW and patient cohorts. In addition, antigens were selected by combining evidence from multiple groups and arrays with prior knowledge from literature and in-house studies. The final panel included 307 protein fragments and 56 full-length proteins. Together, these 363 antigens represented proteins from 315 genes (Supplementary Data 1).

### Prevalent and persistent new-onset autoantibodies emerge with COVID-19

**Fig. 2:**
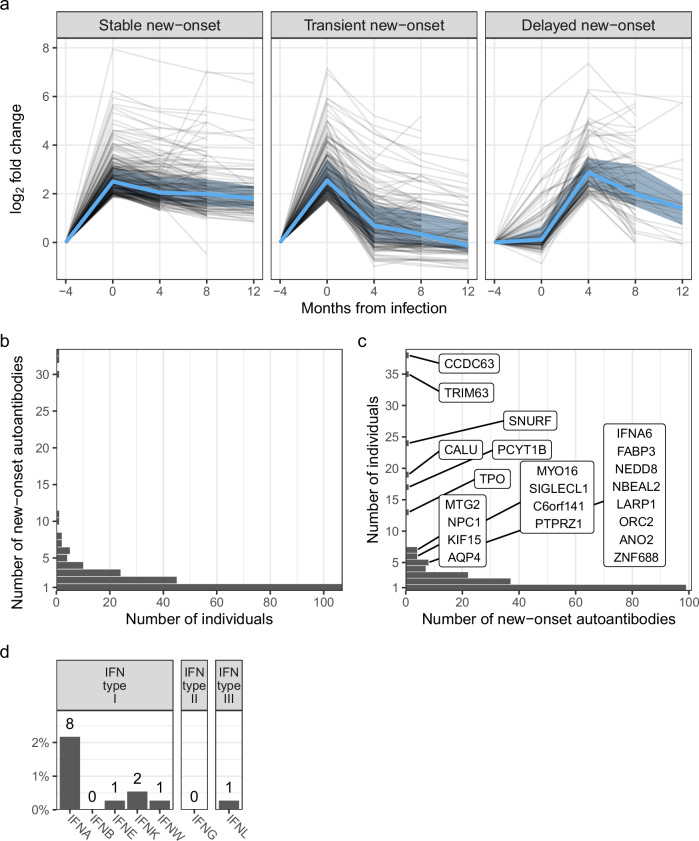
Prevalent and persistent new-onset autoantibodies emerge with COVID-19. **a** Persistence of new-onset autoantibodies across categories. New-onset autoantibodies were categorized by their dynamics: Stable, transient, or delayed. Black lines represent new-onset autoantibody trajectories based on fold change in relation to seronegative baseline. Blue lines and shaded areas represent the median and quartiles, respectively. **b** Distribution of new-onset autoantibodies among baseline seronegative individuals. Bars depict the number of individuals with the indicated number of new-onset autoantibodies. **c** Distribution of new-onset autoantibody prevalence. Bars depict the number of new-onset autoantibodies with the indicated prevalence. The 22 prevalent new-onset autoantibodies with prevalence >1% (number of individuals >4) are indicated with gene names. **d** Prevalence of new-onset autoantibodies toward interferon (IFN) subtypes. Source data are provided as a Source Data file.

For the initial investigation of new-onset autoantibodies, data from the full HCW and patient cohorts were filtered to include individuals with three consecutive samplings before, at, and after seroconversion (*n* = 369: 362 HCW and seven hospitalized patients). Individual autoantibody trajectories were defined by calculating fold change (FC) of autoantibody levels at the two later time points relative to the seronegative baseline. Clustering of all obtained trajectories revealed three distinct categories of new-onset autoantibodies shown in Fig. 2a: stable (*n* = 225), transient (*n* = 177), and delayed (*n* = 103) new-onset autoantibodies. Four additional clusters of relatively unchanging trajectories were detected and not classified as new-onset (Supplementary Fig. 2). The new-onset autoantibody landscape of individuals and antigens with at least one detected new-onset autoantibody (*n* individuals = 204, *n* antigens = 187) is displayed in Supplementary Fig. 3. These autoantigens represent extracellular (*n* = 57) and intracellular (*n* = 119) proteins corresponding to 176 genes as classified in the Human Protein Atlas^32^.

We further examined the persistence of new-onset autoantibodies in the 160 trajectories where 12-month follow-up data was available (stable and transient trajectories in 63 individuals). Persistence, defined as FC ≥2 at 12 months compared to baseline, was observed for the majority of autoantibodies with stable trajectories (95% (78/82)), while only 23% (18/78) of transient new-onset autoantibodies remained elevated. In total, 60% of new-onset autoantibodies remained elevated 12 months after onset.

Further investigation of the trajectories revealed that new-onset autoantibodies were found in 204 of the 369 individuals and that they targeted a total of 187 antigens. Most individuals displayed single new-onset autoantibodies (*n* = 107), but three individuals we found to have 30 to 33 (Fig. 2b). In line with previous reports^12,14,33^, most autoantibodies (99 of 187 detected) were rare and occurred in single individuals (Fig. 2c). The 22 most prevalent new-onset autoantibodies, detected in >1% of the cohort (>4 individuals), were subjected to further analysis (Table 1 and Supplementary Data 2). The corresponding antigens represented both intracellular (*n* = 16, 73%) and extracellular (*n* = 6, 27%) proteins. Autoantibodies which have previously been reported in autoimmune diseases or COVID-19 patients were present among the most prevalent new-onset autoantibodies, including anti-TPO (thyroid peroxidase)^11^, anti-AQP4 (aquaporin-4)^9^ and anti-IFNA^1^ IgG. However, the emergence of these autoantibodies with COVID-19 has not been reported previously. Several of the most prevalent new-onset autoantibodies have, to our knowledge, not been described previously, including the three with the highest prevalence, i.e., anti-CCDC63 (coiled-coil domain-containing protein 63), anti-TRIM63 (E3 ubiquitin-protein ligase TRIM63), and anti-SNURF (SNRPN upstream reading frame protein) IgG. In total, 150 of the 369 baseline seronegative individuals (41%) developed at least one of the 22 most prevalent new-onset autoantibodies.

**Table 1:** New-onset autoantibodies with >1% prevalence

Gene	Protein	Antigen	ENSP	aa	New-onset		Acute onset (%)	Baseline seronegative (%)
					*n*	%		
CCDC63	Coiled-coil domain-containing protein 63	HPRR3070734	ENSP00000312399	155–249	49	10	96	78
SNURF	SNRPN upstream reading frame protein	HPRR4280292	ENSP00000463201	45–69	49	10	76	49
TRIM63	E3 ubiquitin-protein ligase TRIM63	HPRR3760711	ENSP00000363390	182–246	44	9	66	80
CALU	Calumenin	HPRR4180820	ENSP00000249364	74–128	32	6	41	59
TPO	Thyroid peroxidase	HPRR4180385	ENSP00000329869	867–932	22	4	95	59
PCYT1B	Choline-phosphate cytidylyltransferase B	HPRR4320357	ENSP00000368439	329–369	22	4	86	77
MYO16	Unconventional myosin-XVI	HPRR3090005	ENSP00000401633	843–923	18	4	89	33
AQP4	Aquaporin-4	HPRR2140210	ENSP00000372654	253–323	17	3	35	41
NPC1	NPC intracellular cholesterol transporter 1	HPRR620021	ENSP00000269228	448–580	14	3	79	50
NBEAL2	Neurobeachin-like protein 2	HPRR3460329	ENSP00000415034	1865–1939	13	3	100	38
ZNF688	Zinc finger protein 688	HPRR3610292	ENSP00000223459	95–166	12	2	92	42
IFNA6	Interferon alpha-6	HPRR3360040	ENSP00000369558	74–99	12	2	25	42
SIGLECL1	SIGLEC family-like protein 1	HPRR3420383	ENSP00000469601	11–94	10	2	90	60
MTG2	Mitochondrial ribosome-associated GTPase 2	HPRR3300084	ENSP00000359859	317–390	10	2	80	70
NEDD8	NEDD8	HPRR2760190	ENSP00000250495	7–67	10	2	70	50
KIF15	Kinesin-like protein KIF15	HPRR2960531	ENSP00000324020	671–748	10	2	60	70
FABP3	Fatty acid-binding protein, heart	HPRR3890315	ENSP00000362817	30–58	9	2	67	56
ANO2	Anoctamin-2	HPRR3070036	ENSP00000507275	79–167	9	2	56	56
LARP1	La-related protein 1	HPRR3760556	ENSP00000428589	254–329	9	2	44	56
ORC2	Origin recognition complex subunit 2	HPRR2920267	ENSP00000234296	150–224	8	2	75	62
C6orf141	Uncharacterized protein C6orf141	HPRR2660035	ENSP00000434602	5–73	7	1	86	86
PTPRZ1	Receptor-type tyrosine-protein phosphatase zeta	HPRR4170025	ENSP00000377047	441–534	6	1	50	100

New-onset autoantibodies were assessed in 502 individuals (456 HCW and 46 hospitalized COVID-19 patients, after exclusion of outlying or incomplete data).

Considering the demonstrated impact of antibodies targeting interferons (IFNs) in COVID-19^1^, we specifically investigated the prevalence of new-onset autoantibodies across IFN subtypes. In total, we detected new-onset anti-IFN IgG in 10 of 362 baseline seronegative HCW and 1 of the 7 COVID-19 patients that were seronegative at admission. Anti-interferon alpha (IFNA) IgG was predominant (Fig. 2d), and 1 individual had new-onset autoantibodies targeting more than one IFN subtype (IFNA, interferon epsilon (IFNE), and interferon omega (IFNW)).

### Prevalent new-onset autoantibodies in individuals without pre-infectious samples

Next, we aimed to explore associations of the 22 most prevalent new-onset autoantibodies to COVID-19 severity and symptoms post-COVID-19. To include individuals who were seropositive at study inclusion (94 HCW and 39 hospitalized patients), we first developed a model for classification of new-onset based on autoantibody levels at seroconversion and four- and eight-month follow-up. The model used the aggregated categories acute new onset (stable or transient) and delayed new-onset.

This multinomial linear regression (MNL) model was trained on the autoantibody trajectories of the previously assessed baseline seronegative individuals that had an eight-month follow-up sample after seroconversion (*n* individuals = 282; *n* trajectories for training = 6204). Using an 80%/20% training/testing split, we found that the model performed well on the test set and was highly specific although moderately sensitive (specificity = 0.997, sensitivity = 0.667, AUC = 0.83). Applied to the baseline seropositive individuals, the model classified 98 autoantibody trajectories as acute new-onset and 56 as delayed new-onset in 79 individuals (59%) (Supplementary Fig. 4). The high specificity and moderate sensitivity of the model indicates that it may underestimate new-onset autoantibody prevalence in individuals without baseline seronegative samples. Still, the 22 new-onset autoantibodies were significantly more prevalent in individuals without baseline seronegative samples than in individuals with baseline seronegative samples (average prevalence 5.3 and 2.9%, respectively; odds ratio (OR) (95% confidence interval (CI)) = 1.7 (1.3–2.1), *p* = 4 × 10^−6^, *n* trajectories = 11,044). This could indicate increased autoimmune signatures in hospitalized COVID-19 patients.

**Fig. 3:**
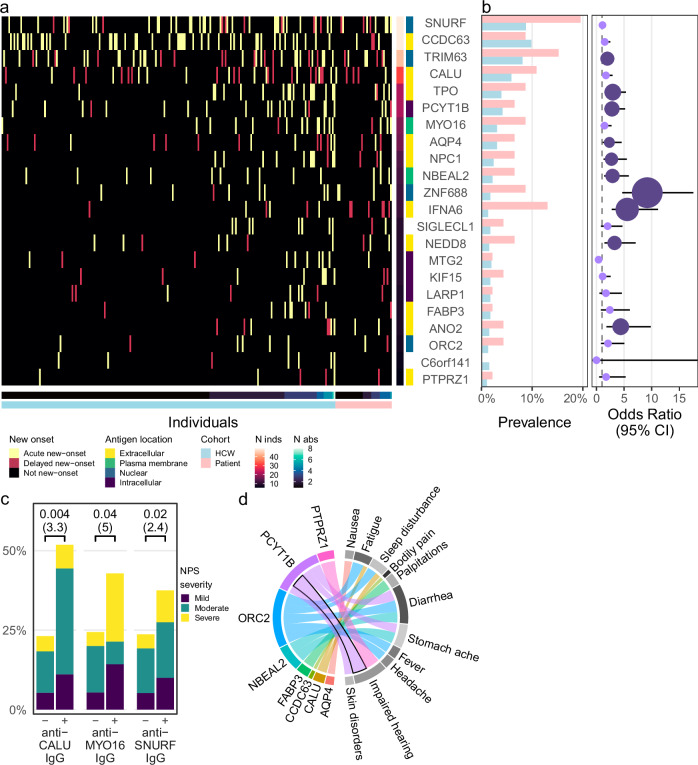
New-onset autoantibodies have increased prevalence in hospitalized COVID-19 patients and are associated with neuropsychiatric symptoms post-COVID-19. **a** Overview of new-onset autoantibodies in healthcare workers (HCW) and hospitalized COVID-19 patients. The 22 prevalent new-onset autoantibodies are grouped by their antigen location. Columns show individuals with at least one prevalent new-onset autoantibody. Cell color represents the new-onset autoantibody category: Acute, delayed, or not new-onset. **b** Hospitalized COVID-19 patients showed increased prevalence of nearly half of the most prevalent new-onset autoantibodies. Bars indicate autoantibody prevalence in patients (pink) and HCW (blue). Points and error bars indicate odds ratio (OR) with 95% confidence interval (CI) based on logistic regression. Point size and color shows magnitude of *q* value (Benjamini–Hochberg). Light purple: *q* > 0.05. Dark purple: *q* ≤ 0.05. *n* HCW = 456, *n* patients = 46. **c** Increased severity of neuropsychiatric symptoms post-COVID-19 was associated with three new-onset autoantibodies. Bars depict proportions of HCW with different severities of neuropsychiatric symptoms post-COVID-19 among autoantibody-positive (+) and negative (−) groups. Brackets indicate *p* values and odds ratios (in parentheses) from proportional odds logistic regression. No adjustment of *p* values was made. **d** Eleven symptoms post-COVID-19 were associated with eight of the most prevalent new-onset autoantibodies. Band colors indicate the new-onset autoantibody. Bandwidths indicate the estimated logit (log odds ratio) of associations with *p* ≤ 0.05 based on logistic regression. The indicated association of PCYT1B and impaired hearing remained significant after Benjamini–Hochberg correction for multiple comparisons (*q* = 0.002). Source data are provided as a Source Data file.

Summarizing the new-onset autoantibody landscape in both seronegative and seropositive baseline individuals, 43% of HCW (*n* = 196/456) and 72% of hospitalized COVID-19 patients (*n* = 33/46) displayed at least one of the most prevalent new-onset autoantibodies (Fig. 3a). As shown in Fig. 3b, 10 of the 22 were significantly more prevalent in the patients than the HCW, regardless of the number of symptoms post-COVID-19 (logistic regression model adjusted for age and sex, Benjamini–Hochberg correction, *q* ≤ 0.05).

### New-onset autoantibodies are associated with neuropsychiatric symptoms post-COVID-19

Next, we investigated whether the most prevalent new-onset autoantibodies were associated with neuropsychiatric symptoms post-COVID-19 in HCW. Using a proportional odds logistic regression model, we identified three new-onset autoantibodies associated with increased odds of higher severity of neuropsychiatric symptoms lasting for at least 2 months post-COVID-19, shown in Fig. 3c; anti-CALU (calumenin), anti-MYO16 (unconventional myosin-XVI), and anti-SNURF IgG (OR (95% CI) = 3.3 (1.4–7.4), 5.0 (1.1–24), 2.4 (1.1–5.1); *p* = 0.004, 0.04, 0.02, respectively). In addition, we asked whether the most prevalent new-onset autoantibodies were associated with other post-COVID-19 symptoms. Using logistic regression, we identified eight autoantibodies associated with 11 other reported moderate or severe symptoms post-COVID-19, with the association between anti-PCYT1B (choline-phosphate cytidylyltransferase B) IgG and impaired hearing being the strongest (OR (95% CI) = 41 (8.3–220), *q* = 0.002; Fig. 3d). Interestingly, there was also a moderate association between autoantibodies towards the muscle protein CCDC63 and muscle and joint pain (OR (95% CI) = 2.3 (1.1–4.9), *p* = 0.03), although this association was not significant after Benjamini–Hochberg correction.

### Anti-SNURF IgG increases after infection and after vaccination

**Fig. 4:**
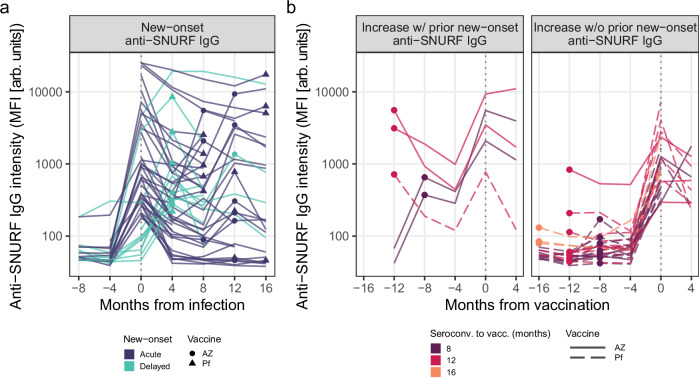
Increasing levels of anti-SNURF IgG are observed both after infection and after vaccination. **a** Antibody trajectories of new-onset anti-SNURF IgG display marked increases at the time of new-onset. Each line depicts the anti-SNURF IgG trajectory of an individual with new-onset anti-SNURF IgG, centered on the time of infection. Line color: mode of new-onset. Points and shape: time of vaccination and vaccine type. **b** Antibody trajectories of individuals with a fourfold increase of anti-SNURF IgG levels at the time of vaccination (vs 4 months prior). Each line depicts the anti-SNURF IgG trajectory centered on the time of first vaccination. Individuals are separated across panels based on whether they had prior new-onset anti-SNURF IgG (emerging after infection). Line color: time between seroconversion and first vaccination. Points: time of seroconversion. Line type: vaccine type. MFI median fluorescent intensity. AZ AstraZeneca. Pf Pfizer/BioNTech. Source data are provided as a Source Data file.

While all 456 HCW that were assessed for new-onset autoantibodies had seroconverted before vaccination, 362 (79%) received their first SARS-CoV-2 vaccine dose during the study period. The majority received the Pfizer/BioNTech vaccine (68%, *n* = 248), 30% received the AstraZeneca vaccine (*n* = 107), and 2% received the Moderna vaccine (*n* = 7). With this in mind, we asked whether any of the 22 most prevalent new-onset autoantibodies not only increase after infection, but also after vaccination. Considering changes at the level of log_2_ FC ≥2, we identified 12 autoantibodies in 45 individuals (Supplementary Fig. 5). Notably, anti-SNURF IgG was the most commonly increasing autoantibody after vaccination (67%; detected in 30 of 45 HCW displaying an increase). As seen in Fig. 4, individuals with as well as without previous new-onset anti-SNURF IgG displayed increases in anti-SNURF IgG at vaccination (Fig. 4b) at comparable levels that of new-onset (Fig. 4a). However, the odds were greater for HCW with previous new-onset anti-SNURF IgG (5 of 28 vs 25 of 332, respectively; OR (95% CI) = 3.4 (1.0 – 9.5), *p* = 0.03, *n* = 360). As four of these five individuals had received the AstraZeneca SARS-CoV-2 vaccine, we investigated whether the odds of autoantibody increase at vaccination was influenced by any interaction effect of previous new-onset autoantibodies and vaccine type and did not find sufficient evidence to support this notion (OR (95% CI) = 10 (0.94–250), *p* = 0.08, *n* = 360).

### Main epitopes of new-onset autoantibodies

Furthermore, we asked what epitopes were targeted by new-onset autoantibodies. To address this question, we epitope mapped eight of the 22 most prevalent new-onset autoantibodies: the highly prevalent anti-CALU, CCDC63, SNURF, and TRIM63 IgG; the previously described anti-IFNA6, ANO2 (anoctamine 2), and TPO IgG; and anti-NPC1 (NPC intracellular cholesterol transporter 1) IgG for which the antigen has sequence overlap with TPO. The epitope mapping was performed on samples from the 142 individuals that had one or more of the eight selected new-onset autoantibodies, using an array of custom-designed 14- and 15-mer peptides with an overlap of 10 to 13 amino acid residues (Supplementary Data 3).

**Fig. 5:**
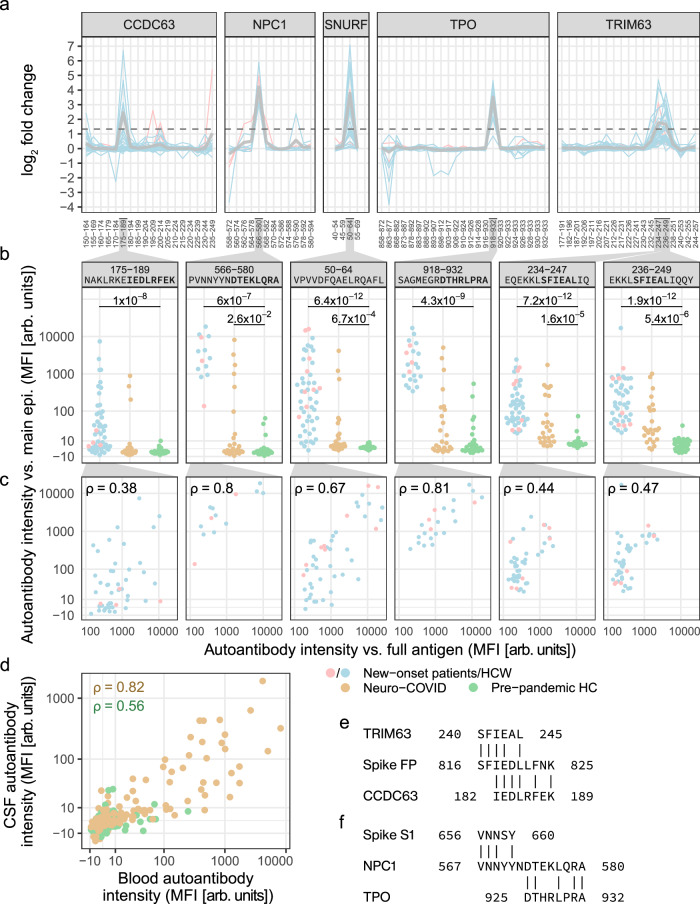
The main epitopes of new-onset autoantibodies are elevated in blood and CSF of an independent cohort with neuro-COVID and align with the SARS-CoV-2 fusion peptide. **a** Epitope mapping revealed the main epitopes of six new-onset autoantibodies. Lines depict epitope profiles (anti-peptide IgG log_2_ fold change (FC)) at new-onset in individuals with the corresponding new-onset autoantibody. Gray lines depict the mean. The dashed line indicates the cutoff for classification as a main epitope (FC ≥2.52). Numbering on the *x*-axis corresponds to the peptide amino acid positions in the full-length protein (Supplementary Data 3). **b** Antibodies against the main epitopes were elevated in an independent cohort of neuro-COVID patients compared to pre-pandemic HCs. Brackets indicate a statistically significant difference to pre-pandemic HC (*q* values ≤ 0.05 from two-sided Mann–Whitney *U* tests with Benjamini–Hochberg correction). The *y*-axis displays signal intensity on the pseudo-log_10_ scale. **c** Correlation of autoantibody intensity against the main epitopes (epi., *y*-axis) and the full antigen (*x*-axis). *ρ* Spearman’s rho. **d** Correlation of autoantibodies against the main epitopes in blood and CSF of neuro-COVID patients and pre-pandemic HCs. Data points correspond to paired blood and CSF samples in neuro-COVID patients (*n* = 23) and HCs (*n* = 21), and the peptides shown in panel (**b**). **e**, **f** Sequence similarity between (**e**) the main epitopes of TRIM63 and CCDC63 to the SARS-CoV-2 Spike fusion peptide, and **f** between the main epitopes of TPO and NPC1 and the C-terminal domain of Spike S1. MFI median fluorescent intensity, HC healthy controls, HCW healthcare workers. Source data are provided as a Source Data file.

Five autoantibodies displayed main epitopes that were common to individuals with the corresponding new-onset autoantibody. The epitopes correspond to the peptides CCDC63|175-189, NPC1|566–580, SNURF|50−64, TPO|918–932, TRIM63|234–247, TRIM63|236–249, and ANO2|135-149. Aside from the main epitopes, other epitopes occurred individually (Fig. 5a). We next investigated the correspondence of autoantibodies targeting the main epitopes and autoantibodies detected against the full antigen. We observed significantly elevated log_2_ FC of autoantibodies against the main epitopes in individuals with vs without the respective new-onset autoantibody, except in anti-ANO2|135-149 IgG which therefore was excluded from further analysis (Supplementary Fig. 6). The correlation of autoantibodies detected using different antigen representations varied from a general correlation, e.g., for the SNURF antigens, to a weak correlation in the CCDC63 antigens (Fig. 5c).

### Validation of autoantibodies against the main epitopes in blood and cerebrospinal fluid

To validate our findings, we analyzed two independent cohorts of pre-pandemic HCs, (*n* = 29) and neuro-COVID patients (*n* = 25) for the presence of autoantibodies against the detected main epitopes. Cohort demographics are presented in Supplementary Table 1. Compared with levels in pre-pandemic HCs, the levels of autoantibodies against the main epitopes were significantly elevated in the individuals with the corresponding new-onset autoantibody (CCDC63|175–189: *q* = 1 × 10^−8^, *f* = 0.9; NPC1|566-580: *q* = 6 × 10^−7^, *f* = 1; SNURF|50-64: *q* = 6.4 × 10^−12^, *f* = 0.97; TPO|918-932: *q* = 4.3 × 10^−9^, *f* = 0.99; TRIM63|234–247: *q* = 7.2 × 10^−12^, *f* = 0.98; TRIM63|236–249: *q* = 1.9 × 10^−12^, *f* = 1). In addition, anti-NPC1|566-580 (*q* = 2.6 × 10^−2^, *f* = 0.68), anti-SNURF|50–64 (*q* = 6.7 × 10^−4^, *f* = 0.77), anti-TRIM63|234–247 (*q* = 1.6 × 10^−5^, *f* = 0.84), and anti-TRIM63|236–249 (*q* = 5.4 × 10^−6^, *f* = 0.86) IgG levels were significantly increased in neuro-COVID patients compared to pre-pandemic HCs. Notably, the only autoantibody for which the pre-pandemic HCs had levels above background was the previously described autoantibody anti-TPO IgG (Fig. 5b).

As we were specifically interested in the possible neurological pathology of autoantibodies, we asked whether epitope-directed autoantibodies could also be found in the cerebrospinal fluid (CSF) of individuals with COVID-19. In the validation cohorts, paired CSF and blood samples were available for 23 neuro-COVID patients and 21 pre-pandemic HCs. As seen in Fig. 5d, epitope-directed autoantibody signals correlated in CSF and blood of both validation cohorts (neuro-COVID: *ρ* = 0.82, *p* < 2.2 × 10^−16^; HCs: *ρ* = 0.56, *p* = 9.5 × 10^−13^).

### The muscle proteins TRIM63 and CCDC63 align with the SARS-CoV-2 fusion peptide

One possible explanation for the emergence of new-onset autoantibodies is molecular mimicry between viral and human proteins. Therefore, we examined any amino acid sequence similarity of the main epitopes and the SARS-CoV-2 Spike glycoprotein^34^ (UniProt accession P0DTC2) using BLAST^35^. We found sequence similarities between the Spike glycoprotein sequence 816-SFIEDLLFNK-825 and TRIM63|234–247 (*E* = 0.022), TRIM63|236-249 (*E* = 0.022), and CCDC63|175–189 (*E* = 0.012) (Fig. 5e). This sequence is proximal to the S2’ cleavage site of Spike protein fusion peptide. Furthermore, we found an alignment of NPC1|566-580 and the Spike S1 C-terminal domain sequence 656-VNNSY-660 (*E* = 0.60), which is exposed at the surface of S1. In addition, NPC1|566–580 and TPO|918–932 share five residues (Fig. 5f).

## Discussion

In the present work, we have characterized the autoantibody response emerging with COVID-19 using proteome-wide autoantibody screening in longitudinal and independent cohorts. As the scope of our study was a proteome-wide analysis of autoantibody repertoires, we searched for autoantibodies towards intracellular as well as extracellular and secreted antigens. Although the pathogenic mechanisms of antibodies towards intracellular antigens remain unclear, they are frequently observed in screening studies and can be of established clinical importance^36^.

While previous studies have indicated the existence of new-onset autoantibodies in COVID-19^15,16^ and in other pulmonary infections^37^, we have systematically charted the temporal dynamics of the emerging self-directed humoral response in COVID-19 and showed that 60% of new-onset autoantibodies remained elevated for at least 12 months after infection. In addition, we have shown that new-onset autoantibody prevalence corresponds to acute disease severity. Taken together, these results indicate that a dysregulated humoral immune response is a marked feature of acute and post-acute COVID-19. Furthermore, our study shows that there is large diversity and interindividual heterogeneity of new-onset autoantibodies in COVID-19, corroborating previous findings in cross-sectional autoantibody studies in health^14^ and disease^33,38^, including COVID-19^12^. The detected new-onset autoantibodies target a wide range of antigens across the proteome, illustrating the breadth of the autoantibody response emerging after COVID-19.

The dynamics of detected new-onset autoantibodies followed three distinct patterns: stable, transient, and delayed onset. The two acute onset types reflect different autoantibody persistence, while delayed onset may reflect other parameters. As 31% (49 of 159) of delayed new-onset autoantibodies emerged in individuals who were vaccinated between seroconversion and the subsequent visit, it is not possible to deconvolve the influence of the events on the onset of these autoantibodies. While the processes behind the remaining 69% are not clear, we speculate that a short time between infection and blood sampling could prevent immediate detection of new-onset autoantibodies despite detection of seroconversion, possibly due to the high sensitivity of the serological assay^28^. Alternatively, delayed autoantibody onset could reflect autoantibody emergence in late affinity maturation. Further work is needed to shed light on the mechanisms underlying the emergence of new-onset autoantibodies.

As anti-IFN IgG is implied in severe COVID-19, we specifically characterized the new-onset anti-IFN IgG response in our study. Considering all IFN subtypes, we found new-onset anti-IFN IgG in 3% of the baseline seronegative cohort, mainly consisting of anti-type I IFN IgG. These results are corroborated by previous studies indicating that anti-IFN IgG in COVID-19 are mainly directed against type I IFNs and that these autoantibodies typically existed prior to COVID-19^1^, although results suggesting new-onset anti-IFN antibodies have also been reported^39^.

Since neurological symptoms after COVID-19 are commonly occurring and often debilitating, we made directed efforts in understanding this group of symptoms post-COVID-19. We found three new-onset autoantibodies associated with increased severity of neuropsychiatric symptoms post-COVID-19: anti-CALU, MYO16, and SNURF IgG. CALU is a membrane-bound or secreted calcium-binding protein mainly expressed in the heart and skeletal muscle. MYO16 is a cytoplasmic unconventional myosin with enhanced brain expression, and may be involved in the extension of neuronal membrane processes^40^. SNURF is a small (71 aa) nuclear protein of unknown function, primarily expressed in brain and muscle tissues and cardiomyocytes. In addition, we found associations of 11 self-assessed non-neurological symptoms post-COVID-19 to 8 of the most prevalent new-onset autoantibodies. However, these associations were tentative, with only the association of anti-PCYT1B IgG and impaired hearing remaining significant after FDR correction.

Our epitope mapping identified the main epitopes of five autoantigens. These epitopes showed a marked increase in reactivity after infection. Among these, the main epitopes of TRIM63 and CCDC63 displayed a sequence alignment to the fusion peptide of the SARS-CoV-2 spike glycoprotein. While not sufficient in isolation, the high post-infectious reactivity to these autoantigens combined with their sequence alignment to a viral protein are together consistent with a molecular mechanism invoking molecular mimicry. The fusion peptide is crucial for viral entry into the host cell and is highly conserved across the family of coronaviruses. Furthermore, it is accessible to antibody binding in the post fusion state, after engaging the ACE2 receptor^41^. Concordantly, two studies have independently found that human antibodies that broadly neutralize coronaviruses are targeting the fusion peptide^41,42^. This raises the possibility that the observed mimicry also might be found in infections with other coronaviruses. To our knowledge, however, these antigens have not previously been investigated in this context. Notably, similar evidence of molecular mimicry between sorting nexin-8 (SNX8) and the SARS-CoV-2 Nucleocapsid protein has recently been reported in multisystem inflammatory syndrome in children following COVID-19^43^. We did, however, not find any evidence suggestive of molecular mimicry with Nucleocapsid in our study.

With the independent cohort, we validated the presence of anti-TRIM63|234–247, and anti-TRIM63|236–249 IgG in patients with neuro-COVID but not in pre-pandemic HCs. In the discovery cohort, anti-TRIM63 IgG prevalence was increased in hospitalized COVID-19 patients. In addition, we observed a moderate association between anti-CCDC63 IgG and self-reported muscle and joint pain. Taken together, there is evidence suggestive of molecular mimicry between the immunologically important Spike protein fusion peptide and the muscle proteins TRIM63 and CCDC63 alongside associations with muscular symptoms post-COVID-19 and COVID-19 severity.

Despite not showing any sequence similarity with SARS-CoV-2, anti-SNURF IgG emerged in 9% of HCW (*n* = 40) and 20% of hospitalized COVID-19 patients (*n* = 9). Furthermore, antibodies against the main epitope of SNURF (aa 50–64) were validated in the blood and CSF of patients with neuro-COVID (compared to pre-pandemic HCs). Our detected correlation of autoantibody levels in the blood and CSF corroborates findings of blood-brain barrier disruption in patients with cognitive impairment post-COVID-19^44^. It is worth noting that SNURF, like the other two most prevalent autoantibody targets TRIM63 and CCDC63, is expressed in heart and skeletal muscle. In addition, SNURF is expressed in several other tissues, including the brain, corroborating the association of anti-SNURF IgG and neuropsychiatric symptoms post-COVID-19. Like CCDC63 and many classical autoantigens, SNURF is located in the nucleus, indicating that epitope spreading may be the source of anti-SNURF IgG^45^. Furthermore, anti-SNURF IgG was by far the most common autoantibody increasing at vaccination. While the data did not sufficiently support an interaction effect of previous new-onset anti-SNURF IgG and vaccine type on anti-SNURF IgG increase at vaccination, this could be due to small sample sizes. However, a recent study reported that autoantibodies remained stable after vaccination with SARS-CoV-2 mRNA vaccines and were not elevated in patients with vaccine-associated myocarditis^16^. Similarly, other studies reported that established autoantibodies do not increase after vaccination^46,47^. In line with this, our study does not provide any evidence linking the herein-detected autoantibodies with any adverse effects following vaccination.

Previous longitudinal studies have reported preexisting but not new-onset autoantibodies to the clinically important autoantigen TPO in COVID-19 patients^15^. In contrast, we found new-onset anti-TPO IgG in 4% (*n* = 22) of the longitudinal cohort, with a higher prevalence in hospitalized COVID-19 patients than in HCW with mild to moderate disease. In addition, we showed that new-onset anti-TPO IgG co-occurred with new-onset anti-NPC1 IgG in 10 of 22 cases, and anti-NPC1 IgG appeared without anti-TPO IgG in only four individuals. Subsequent epitope mapping and sequence alignment revealed that the main epitopes of TPO and NPC1, which display elevated autoantibody levels after infection, have a sequence similarity containing five identical residues. In addition, the N-terminal residues of the main epitope of NPC1 align with the amino acid sequence 656-VNNSY-660 at the C-terminal domain of the Spike glycoprotein S1 subunit. Together, this raises the possibility of molecular mimicry of NPC1 and Spike S1, with epitope spreading through molecular linkage yielding anti-TPO IgG^45^. Although anti-TPO IgG benignly occurs in around 10% of the healthy population^48^, our discovery of new-onset antithyroid antibodies emerging with mild to severe COVID-19 is concerning given that large epidemiological studies have shown increased incidence of autoimmune and autoinflammatory disorders, including autoimmune thyroid diseases, following COVID-19^49,50^. However, we did not detect any anti-TPO IgG increases at vaccination. To our knowledge, anti-NPC1 IgG has not previously been identified following SARS-CoV-2 infection. In contrast, the NPC1 protein has previously been suggested^51,52^ and recently reported^53^ as an alternative and inhibitable infective entry point of SARS-CoV-2. Together, these findings warrant further research on NPC1, anti-NPC1 IgG, and antithyroid antibodies in COVID-19.

Comparing autoantibody prevalence between studies is an interesting but challenging task due to differences in antigen representation and response criteria. However, we note that only one of the five epitope-mapped autoantibodies, anti-TPO IgG, have been previously reported in proteome-scale autoantibody studies of COVID-19, with a reported prevalence ranging from 0^12,16,27,43,54^ to 2%^15^. These differences may arise from different collections of antigen representations varying by, e.g., length, proteome coverage, and post-translational modifications, thus representing different sets of antigenic space. Similarly, the design of the analytical approach, e.g., longitudinal or cross-sectional study design, control sample set, and response criteria, can contribute to differences between studies.

Validation of detected autoantibodies is an important matter requiring careful consideration. Here, we performed new-onset autoantibody discovery using protein fragments and thoroughly validated them using 14- and 15-mer peptides, as this approach enables detection of the specific epitopes of interest. Additional validation on full-length proteins or clinical assays has the potential to aid clinical translation. This can, however, be confounded by the larger epitope space of larger antigens unless an experimental step to enrich the antibodies of interest is used, i.e., antibody enrichment on the peptide representing the epitope of interest. As antibody enrichment experiments require more sample volume than that which is available for the present cohorts, this additional validation is left for future studies.

Certainly, our study has limitations. Although powerful, the Proteome-wide arrays do not detect autoantibodies towards epitopes that are not covered by the protein fragments on the arrays, or conformational epitopes. Furthermore, as symptoms post-COVID-19 were of moderate prevalence and obtained by self-assessment, and their pre-pandemic prevalence in the cohort is not known, clinical associations are limited and require validation in cohorts where symptoms post-COVID-19 are more prevalent and have been assessed by a clinician. Moreover, as our controls were healthy and without other infections, the detected new-onset autoantibodies might also develop after other infections than SARS-CoV-2 infection. In addition, the functional properties of the presented autoantibodies remain tentative, and cross-reactivity should be further evaluated in future work using competitive assays.

In summary, our study shows that new-onset autoantibodies are prevalent and persistent following mild to severe COVID-19 and correspond to disease severity. Furthermore, some are found to be associated with neuropsychiatric symptoms post-COVID-19 and could be detected in both plasma and CSF of patients with neuro-COVID. In addition, we demonstrate that anti-TRIM63 and anti-CCDC63 IgG develop in 10% of the study cohort and reveal their main epitopes using epitope mapping. The main epitopes display sequence similarities indicative of molecular mimicry with the highly conserved fusion peptide of the SARS-CoV-2 Spike glycoprotein, which is essential for viral entry and the target of broadly neutralizing antibodies. Conversely, anti-SNURF IgG is highly prevalent without evidence of molecular mimicry, which may indicate epitope spreading to nuclear antigens. Our work reveals the complexity of the autoantibody repertoire that emerges with COVID-19 and shows its potential impact on the course of acute viral infection and post-viral syndromes. This provides a strong rationale for further exploration of new-onset autoantibody repertoires in other infectious diseases, as well as for continued investigation of the herein presented new-onset autoantibodies.

## Methods

### Study cohorts

The COMMUNITY study is an ongoing longitudinal study which enrolled 2149 HCW and 118 COVID-19 patients admitted to Danderyd Hospital, Stockholm, Sweden, between April and May/June 2020 (HCW/patients)^29–31^. The cohort is followed with blood sampling every four months and we assess anti-SARS-CoV-2 IgG using a multiplex bead array of SARS-CoV-2 proteins^28^. In addition, HCW reported symptoms post-COVID-19 through electronic self-assessment forms at selected visits, including visits 3 to 5. The symptoms for self-assessment were anxiety, brain fatigue, impaired concentration, cough, depressed mood, diarrhea, dyspnea, dizziness, fatigue, fever, hair loss, headache, impaired hearing, impaired memory, ageusia, muscle/joint pain, nausea, numbness, anosmia, palpitations, skin disorders, sleep disturbance, and stomach ache. Symptom severity was graded as mild, moderate, or severe. Neuropsychiatric symptoms were defined as reporting one or several of anxiety, brain fatigue, impaired concentration, depressed mood, and impaired memory. The disease severity of the hospitalized patients ranged from mild to severe. Disease severity and comorbidities are presented in Supplementary Table 1. At the start of the study in April 2020, all patients currently hospitalized with confirmed COVID-19 and all employees were eligible for inclusion, with no exclusion criteria.

In the present study, we retrospectively considered visits 1 to 5, i.e., May 2020 to September 2021. We selected a subgroup of 478 HCW and 47 hospitalized COVID-19 patients based on previous serological results and reported symptoms post-COVID^29–31^. HCW were selected in two steps. First, we selected HCW who were seronegative for anti-SARS-CoV-2 IgG at the first visit in Apr-May 2020, seroconverted prior to vaccination, and had participated in the visits immediately before and after seroconversion (*n* = 381). Second, we selected HCW who were seropositive at the first visit, had participated in all of the four subsequent visits, and had reported several symptoms post-COVID-19 (*n* = 97). Patients were selected based on participation in all of the first four follow-up visits (*n* = 47). In total, 525 individuals were selected, with an average of 4.8 samples per individual, yielding 2532 samples for autoantibody analysis. Demographics are presented in Supplementary Table 1.

Ethical approval for the COMMUNITY study was obtained from the Swedish Ethical Review Authority (Nos. 2020-01653 and 2021-04113). All healthcare workers left written informed consent for study participation. For the patients, oral informed consent was obtained instead of written informed consent due to the risk of contagion. In the case of incapacity, informed consent was obtained from patients’ next of kin. Oral informed consent was recorded in each patient’s medical record as well as in a separate file held by the responsible researcher. The use of oral consent was approved by the Swedish Ethical Review Authority.

The pre-pandemic healthy control group was university employees and students who had not received psychiatric care during their lifetime and were recruited in the timeframe April 2014–April 2017 as healthy controls for the Uppsala Psychiatric Patient Samples (UPP) cohort. Ethical approval for the collection of CSF was acquired by the Regional Ethical Review Board in Uppsala, Sweden (Nos. 2012/081 and 2014/148). Oral and written consent was obtained from all controls. All available controls with matched CSF and serum were selected for this study. Participants underwent a clinical health examination, including blood pressure and body mass index (BMI), and answered questionnaires on socio-demographics, medical history, heredity, and current medication, as well as an interview to evaluate any psychiatric symptoms. While no controls had ongoing symptoms that required specialist psychiatric care, the frequency of prior or ongoing mild and subclinical states of psychiatric conditions was higher than expected. All samples were acquired pre-pandemic. No other exclusion criteria were applied^55^. Demographics are presented in Supplementary Table 1.

The neuro-COVID population has been described previously^56,57^. Patients were prospectively included between April 2020 and June 2021. Inclusion was based on a positive PCR for SARS-CoV-2 in upper and/or lower airway samples, and at least one new-onset neurological symptom and presence of anti-SARS-CoV-2 IgG in serum, or typical COVID-19 symptoms in combination with pulmonary ground-glass opacities and consolidations on computed tomography scan of the thorax. Patients with previous central nervous system insults were excluded. One patient had a previous cerebrovascular insult. Clinical neurological evaluation was performed by an experienced neurologist. All lumbar punctures were performed as part of the clinical routine for neurological investigations. Neurological manifestations at the time of lumbar puncture included cranial nerve affection, central or peripheral paralysis, extrapyramidal, sensory symptoms, altered mental status including confusion, encephalopathy, and reduced level of consciousness. All patients were hospitalized. The disease severity at the lumbar puncture was moderate to severe. Disease severity, comorbidities, and other demographics are presented in Supplementary Table 1. Written informed consent was obtained from each patient, or next-of-kin if a patient was unable to give consent.

The collection and analysis of neuro-COVID and healthy control samples was approved by the Swedish Ethical Review Authority (No. 2017-043 with amendments 2019-00169, 2020-01623, 2020-02719, 2020-05730, 2021-01469, and 2020-01883; and No. 2022-00526-01). The Declaration of Helsinki and its subsequent revisions were followed.

### SARS-CoV-2 serology

Serological classifications for sample selection were obtained from previous studies of the cohorts^29–31^. For increased resolution in the upper ranges of the data, samples were re-analyzed at a higher dilution (1:5000 vs 1:50) while otherwise following the same procedure^28^.

### Planar arrays

Initial exploration of autoantibody repertoires was performed using two sets of in-house developed protein arrays. The Proteome-wide planar array contains 42,000 protein fragments from the Human Protein Atlas (proteinatlas.org) that represent 18,000 proteins and cover ~40% of the amino acid residues of the human proteome^33,58^. The Secretome array contains 1522 full-length secreted or extracellular proteins from 1482 genes representing 58% of the human secretome, i.e., the proteins secreted by human cells^32^. The experimental procedure has been described previously^14^. In brief, plasma samples were pooled within the described groups and diluted 1:100 in before applying them to planar arrays. After incubation and washes, fluorescently labeled secondary detection antibodies were applied to the array for detection of autoantibody binding and detection of array microspots. Readout was performed in a microarray scanner, where the red channel readout corresponds to autoantibodies and the green channel readout to array microspots. Grid alignment and data acquisition from the scanned images was performed using GenePix Pro 5.1. Selected protein fragments are available on reasonable request.

### Bead arrays

Bead arrays were used for the investigation of new-onset autoantibodies in the COMMUNITY cohort and for epitope mapping in the COMMUNITY and validation cohorts. Bead array construction and assays were performed as previously described^14,59^. For an exploration of new-onset autoantibodies, the coupled antigens had been selected from planar arrays as described below. For epitope mapping, the coupled antigens were 93 custom-synthesized biotinylated peptides of 14 to 15 amino acid residues (GenScript Biotech). Assay readout was performed using Luminex FLEXMAP 3D® instruments with xPONENT® software (Luminex Corp.) and responses recorded as median fluorescent intensity (MFI), in arbitrary units.

### Analysis of planar array data

Planar array data were processed as previously described^33^.

Proteome-wide array data were analyzed per subarray. Spots that were flagged in image acquisition, that were smaller than 30 pixels, or that had a green channel signal lower than 4 SD above the mean local background were removed. Duplicate spots were deduplicated by selecting the spot with highest signal in the green channel. Finally, red channel data were *Z-*scored, and antigens with *Z* ≥ 12 were classified as reactive.

Secretome array data was analyzed per subarray. Microarray spots that were flagged in image acquisition, that were smaller than 40 pixels, or that had a green channel signal at or below the local background level were removed. Duplicate spots were filtered if their CV exceeded 50, and remaining spot pairs were deduplicated by taking the mean. Finally, local background was subtracted from red channel data and resulting values were *Z-*scored. Antigens with *Z* ≥ 8 were classified as reactive.

*Z-*scored planar array data were used for the selection of antigens for further investigation in the full HCW and hospitalized patient cohorts. First, antigens reactive in single samples on single arrays were selected. Second, lowered selection thresholds and detection in multiple samples were used to diversify the selection. The following selection criteria were used: protein fragments meeting *Z* ≥ 8 in multiple samples; full-length proteins meeting *Z* ≥ 4 in multiple samples; protein fragments and full-length proteins meeting *Z* ≥ 4 in single or multiple samples on both array types. Third, antigens from the literature and in-house studies were selected: protein fragments noted in both the literature and in-house studies; protein fragments meeting *Z* ≥ 8 and noted in either the literature or in-house studies; full-length proteins noted in multiple publications; full-length proteins meeting *Z* ≥ 2 and noted in the literature; handpicked protein fragments and full-length proteins noted in either the literature or in-house studies. Selected antigens and their matching selection criteria are listed in Supplementary Data 1.

### Classification of new-onset autoantibodies in individuals with a seronegative baseline sample

New-onset autoantibodies were classified by applying Partitioning Around Medoids (PAM) clustering^60^ to the bead array data of baseline seronegative individuals. The approach is documented in the companion R package abtract^61^.

The log_2_ FC of each autoantibody trajectory was computed relative to the most recent seronegative sample. Individuals with autoantibody data at seroconversion and the samplings immediately before and after seroconversion were considered for PAM clustering (*n* = 374). Principal component analysis (PCA) was conducted on these data to identify and exclude outlying individuals with an increase in most autoantibody trajectories at seroconversion (*n* = 5, Supplementary Fig. 7a). Outliers were defined as PC1 ≥ mean + 3 × SD of **PC1** (PC1 ≥8.85). To reduce the running time of the model, trajectories that never exceed background MFI levels were pre-classified as not new-onset. In each antigen class (protein fragments and full-length proteins), background MFI level was defined as robust *Z-*score = 3 in the seronegative samples from September 2020 and January 2021 from the 40 individuals that seroconverted in May 2021 (MFI_fragment_ ≥73, MFI_full-length_ ≥322; *n* filtered trajectories = 619894/910041 (68%)).

The PAM clustering was performed with the custom cosine × euclidean distance metric. To evaluate the parameter *k* (number of clusters), the PAM clustering was run with *k* ranging from 1 to 20, each with 10 random starts of the clustering algorithm. Using the silhouette method, the optimal cluster number was determined to be 7. Based on a median log_2_ FC ≥2, clusters 5, 6, and 7 were classified as new-onset.

### Classification of new-onset autoantibodies in individuals without any seronegative baseline sample

New-onset autoantibodies were assessed in individuals without seronegative baseline samples using multinomial linear regression (MNL)^62,63^.

The MNL model was built using classifications and autoantibody trajectories of the 22 prevalent new-onset autoantibodies in the baseline seronegative individuals that had follow-up samples at 4 and 8 months after seroconversion. The MNL model specification was ${\bf{New}}{\boldsymbol{\hbox{-}}}{\bf{onset\; type}}\, \sim \,{\log }_{10}{{\bf{MFI}}}_{t\left(0\right)}+{{\bf{FC}}}_{t\left(4\right)}+{{\bf{FC}}}_{t\left(8\right)}$, where *t* is months from seroconversion, and **FC** is fold change relative to time of seroconversion (*t*(0)). The trajectories were split into training (0.8) and testing (0.2) sets using randomized stratified sampling on the trajectory outcome (target antigen and simplified new-onset classification; acute, delayed, or not new-onset autoantibody). The MNL model was trained using repeated stratified *K-*fold cross-validation (repeats = 10, *K* = 5, stratification on trajectory outcome). Grid search was used to optimize the penalizing decay parameter, which was set to 0 based on model accuracy.

Individuals that did not have seronegative baseline samples, but that did have autoantibody data at seroconversion and the two samplings immediately after seroconversion, were considered for MNL classification (*n* = 135). PCA was used to exclude outlying individuals with an increase in most autoantibody trajectories in the considered time points (*n* = 2, Supplementary Fig. 7b). Outliers were defined as PC1 ≥ mean + 3 × SD of **PC1** (PC1 ≥2.1). Trajectories of the 22 prevalent new-onset autoantibodies in the remaining 133 individuals were classified using the MNL model (*n* trajectories = 2926).

### Annotation of antigen location

Antigen location was determined based on Cellular Component Gene Ontology (GO) terms for each corresponding gene^64,65^. The GO terms were simplified using the Generic GO subset^66^ and further subdivided into broad location categories as detailed in Supplementary Table 3. For antigens with multiple locations, the annotation was selected in the following order: Extracellular > Plasma membrane > Nuclear > Intracellular. Antigens may lack annotation of location if no parent terms are included in the GO subset.

### Association of new-onset autoantibodies with symptoms post-COVID-19

Association of new-onset autoantibodies with symptoms post-COVID-19 was performed using data from HCW displaying one or more of the 22 prevalent new-onset autoantibodies, or none (*n* = 403). Among these, neuropsychiatric symptoms were defined as reporting anxiety, brain fatigue, difficulties concentrating, depressive disorders, or impaired memory that lasted for at least 2 months after COVID-19 (*n* = 384). The highest reported severity was used (severe = 20 (5%); moderate = 58 (15%); mild = 23 (6%); no neuropsychiatric sx = 283 (74%)). For other symptoms post-COVID-19, reported mild symptoms were excluded (Supplementary Table 4).

### Identification of main epitopes

The peptide bead array was used for epitope mapping. Data were acquired from 142 baseline samples and 150 samples after autoantibody onset in the 142 individuals who had one or more new-onset autoantibodies whose antigens were represented on the peptide bead array. (Eight individuals had both acute and delayed new-onset autoantibodies, increasing the number of samples after autoantibody onset.) The FC was computed relative to baseline, both for beads with coupled peptides as well as the negative control bead with coupled biotin. To adjust for individual background levels, the FC of biotin was subtracted from the FC of the peptides, and the median was set to 1. Any resulting negative values (*n* = 2) were imputed with the smallest positive value present (0.07).

Main epitopes were defined in individuals having the corresponding new-onset autoantibody by taking the mean FC of each peptide. Peptides, where the mean exceeded the background FC cutoff, were defined as main epitopes that were common across individuals. The background FC cutoff was defined as mean + 5 × SD of the FC of the negative biotin control (FC ≥ 2.52).

### Sequence alignment

Sequence alignment was performed using NCBI BLASTP adjusted for short input sequences: *E* < 20 000, word size ≥3, PAM30 substitution matrix, gap costs 7/2, no compositional adjustment, and no filters or masks.

### Statistical analysis

Correlations were performed using Spearman correlation. Group differences of continuous variables were investigated using the Mann–Whitney *U*-test. Binary variables were investigated using logistic regression, categorical variables using multinomial logistic regression, and ordinal variables using proportional odds logistic regression. Regression models used correction for age and sex in group comparisons. False discovery rate correction was performed using the Benjamini–Hochberg procedure, and resulting values are reported as *q* values. All statistical tests were two-sided. All measurements were taken from distinct samples.

All data analysis and statistical analysis was performed in RStudio with R 4.1.2 and 4.2.1 and R packages tidyverse, lubridate, rlang, scales, knitr, pander, httr2, readxl, rstatix, proxy, cluster, caret, MLmetrics, MASS, broom, Omixer, ggpubr, ragg, patchwork, cowplot, GGally, ggsignif, ggfortify, ggdist, ggbeeswarm, ggrepel, ComplexHeatmap, and circlize.

### Reporting summary

Further information on research design is available in the Nature Portfolio Reporting Summary linked to this article.

## Supplementary information

Supplementary Information

Description of Additional Supplementary Files

Supplementary Data 1

Supplementary Data 2

Supplementary Data 3

Reporting Summary

## Source data

Source Data

Transparent Peer Review file

## Data Availability

The antibody repertoire data generated in this study have been registered in the SciLifeLab Data Repository under accession code 26318929. The antibody repertoire data are available under restricted access due to data privacy laws. Access can be obtained by researchers who meet the specified criteria by submitting a request through the Data Repository^67^. The processed antibody repertoire data are provided in the Source Data file. The self-reported post-COVID-19 symptom data are protected and are not available due to data privacy laws. Access requests can be initiated by email to the corresponding author with an approximate timeframe to reply of 4 weeks. The processed post-COVID-19 symptom data were provided in the Supplementary Information and Source Data file. Source data are provided with this paper.

## Code availability

The abtract package for antibody trajectory clustering, implemented in R 4.4.0, is available on GitHub at https://github.com/jernbom/abtract. The version relevant to this work, v0.1.0, is archived on Zenodo^61^.
