# Supplementary material for: Prevalent and persistent new-onset autoantibodies in mild to severe COVID-19
Source: Nat Commun. 2024 Oct 17;15:8941. doi: 10.1038/s41467-024-53356-5 (PMC11484904; doi:10.1038/s41467-024-53356-5)
Supplement: Supplementary file 1 — Supplementary Information [file 41467_2024_53356_MOESM1_ESM.pdf]

Prevalent and persistent new-onset autoantibodies in mild to severe COVID-19

Supplementary information

August F. Jernbom[1], Lovisa Skoglund[1], Elisa Pin[1], Ronald Sjöberg[1], Hanna Tegel[2], Sophia Hober[2], Elham Rostami[3,4], Annica Rasmusson[5], Janet L. Cunningham[5], Sebastian Havervall[6], Charlotte Thålin[6], Anna Månberg[1], Peter Nilsson[1]

[1] Division of Affinity Proteomics, Department of Protein Science, SciLifeLab, KTH Royal Institute of Technology, Stockholm, Sweden

[2] Division of Protein Technology, Department of Protein Science, KTH Royal Institute of Technology, Stockholm, Sweden

[3] Section of Neurosurgery, Department of Medical Sciences, Uppsala University Hospital, Uppsala, Sweden

[4] Department of Neuroscience, Karolinska Institutet, Stockholm, Sweden

[5] Department of Medical Sciences, Psychiatry, Uppsala University, Uppsala, Sweden

[6] Department of Clinical Sciences, Karolinska Institutet, Danderyd Hospital, Stockholm, Sweden

Corresponding author:
August F. Jernbom
august.jernbom@scilifelab.se
augustfa@kth.se

Supplementary Tables

Supplementary Table 1 | Cohort demographics.

	Discovery		Validation	
	HCW	Hosp. patients	Neuro-COVID	Pre-pan-demic HC
***N* individuals**	478	47	25	29
**Age [years, mean (SD)]**	45 (11)	57 (13)	62 (16)	24 (6)
**Sex [*N* (%)]**				
**F**	416 (87%)	16 (34%)	8 (32%)	26 (90%)
**M**	62 (13%)	31 (66%)	16 (64%)	3 (10%)
**Missing data**	0 (0%)	0 (0%)	1 (4%)	0 (0%)
***N* sera**	2297	235	25	29
**per individual [mean (SD)]**	4.8 (0.5)	5 (0)	1 (0)	1 (0)
***N* CSF**	0	0	21	23
**Time of seroconversion [*N* (%)]**				
**May-20**	96 (20%)	40 (85%)		
**Sep-20**	109 (23%)	7 (15%)		
**Jan-21**	233 (49%)	0 (0%)		
**May-21**	40 (8%)	0 (0%)		
**Self-rep. sympt. post-COVID-19 [*N* (%)]**				
**Any**				
**No**	184 (38%)			
**Yes**	294 (62%)			
**Neuropsychiatric**				
**Mild**	29 (6%)			
**Moderate**	83 (17%)			
**Severe**	25 (5%)			
**None**	341 (71%)			
**Respiratory support [*N* (%)]**				
**None**		17 (36%)	12 (48%)	
**Oxygen on nasal cannula or mask**		25 (54%)	5 (20%)	
**Noninvasive respiratory support**		2 (4%)	1 (4%)	
**Intubation**		3 (6%)	5 (20%)	
**Missing data**		0 (0%)	2 (8%)	
**30-day mortality**		0	0	
**New-onset neurological manifestations**		0 (0%)	25 (100%)	
**Preexisting comorbidities**				
**Diabetes**		8 (17%)	4 (16%)	
**Hypertension**		17 (36%)	10 (40%)	
**Cardiovascular disease**		4 (9%)	6 (24%)	
**Cerebrovascular disease**		3 (6%)	1 (4%)	
**Chronic lung disease**		1 (2%)	2 (8%)	
**Asthma**		11 (23%)	1 (4%)	
**Chronic kidney disease**		4 (9%)	1 (4%)	
**Active cancer**		0 (0%)	0 (0%)	
**Neurologic disease**		2 (4%)	0 (0%)	
**Missing data**		1 (2%)	2 (8%)	

Supplementary Table 2 | Groups of HCW and COVID-19 patients analyzed on planar arrays. All samples were anti-Spike IgG positive. Preexisting comorbidities are listed in Supplementary Table 1.

HCW group	Definition
Cardiopulmonary symptoms	Self-reported long-term symptoms >10 months post COVID-19, with ongoing cardiopulmonary symptoms defined as palpitations and shortness at breath at visit 4.
Fever, fatigue, and muscle pain	Self-reported long-term symptoms >8 months post COVID-19, with ongoing general symptoms defined as fever, fatigue, and muscle pain at visit 4.
New diagnoses after COVID-19	Self-reported long-term symptoms >2 months post COVID-19, newly diagnosed with conditions such as postural orthostatic tachycardia syndrome, polymyalgia rheumatica, post-acute sequelae of SARS-CoV-2 infection, or hyperparathyroidism.
Cognitive and neurological symptoms	Self-reported long-term symptoms >8 months post COVID-19, with ongoing cognitive and/or neurological symptoms defined as brain fatigue, memory impairment, concentration difficulties, dizziness, and headache at visit 4.
Dermatological symptoms	Self-reported long-term symptoms >6 months post COVID-19, with ongoing moderate to severe skin problems.
Anosmia or ageusia	Self-reported long-term symptoms >12 months post COVID-19, with ongoing severe symptoms of anosmia and ageusia.
Severe post-COVID-19 symptoms	At least six moderate to severe self-reported ongoing long-term symptoms.
Moderate COVID-19 with lasting symptoms	Self-reported severe COVID-19 and with at least six different self-reported long-term symptoms.
**Hospitalized COVID-19 patient group**	
Critical COVID-19 (male)	Males without any preexisting comorbidities, where the majority received care at the intensive care unit due to COVID-19. 30-day mortality within the group was 50%.
Severe COVID-19 (male)	Males without any preexisting comorbidities, where the majority received care at the intermediate care unit due to COVID-19. 30-day mortality within the group was 25%.
Severe COVID-19 (female)	Females without any preexisting comorbidities, where the majority received care at the intermediate care unit due to COVID-19. 30-day mortality within the group was 25%.
Severe COVID-19 with comorbidities	50% females. Two patients with multiple sclerosis with ongoing immunosuppressive treatment, one patient with Guillan-Barré secondary to COVID-19 and one patient with hypertension. 30-day mortality within the group was 25%.

Supplementary Table 3 | Location categories of the Generic GO term subset.

GO ID	Name	Annotation
GO:0005576	extracellular region	Extracellular
GO:0005615	extracellular space	Extracellular
GO:0005929	cilium	Extracellular
GO:0030312	external encapsulating structure	Extracellular
GO:0031012	extracellular matrix	Extracellular
GO:0005618	cell wall	Plasma membrane
GO:0005886	plasma membrane	Plasma membrane
GO:0000228	nuclear chromosome	Nuclear
GO:0005634	nucleus	Nuclear
GO:0005635	nuclear envelope	Nuclear
GO:0005654	nucleoplasm	Nuclear
GO:0005694	chromosome	Nuclear
GO:0005730	nucleolus	Nuclear
GO:0005739	mitochondrion	Intracellular
GO:0005764	lysosome	Intracellular
GO:0005768	endosome	Intracellular
GO:0005773	vacuole	Intracellular
GO:0005777	peroxisome	Intracellular
GO:0005783	endoplasmic reticulum	Intracellular
GO:0005794	Golgi apparatus	Intracellular
GO:0005811	lipid droplet	Intracellular
GO:0005815	microtubule organizing center	Intracellular
GO:0005829	cytosol	Intracellular
GO:0005840	ribosome	Intracellular
GO:0005856	cytoskeleton	Intracellular
GO:0009536	plastid	Intracellular
GO:0009579	thylakoid	Intracellular
GO:0031410	cytoplasmic vesicle	Intracellular
GO:0043226	organelle	Intracellular

Supplementary Table 4 | Self-reported symptoms post-COVID-19.

Prevalence of self-reported moderate or severe symptoms among HCW. Data pertaining to Figure 3d. Self-reported mild symptoms were excluded.

**Symptom post-COVID-19**	**Moderate or severe [*n*(%)]**	**Not present [*n*(%)]**
Cough	46 (11)	329 (82)
Diarrhea	16 (4)	374 (93)
Dyspnea	57 (14)	321 (80)
Dizziness	28 (7)	356 (88)
Fatigue	144 (36)	222 (55)
Fever	24 (6)	372 (92)
Hair loss	37 (9)	351 (87)
Headache	78 (19)	301 (75)
Impaired hearing	14 (3)	384 (95)
Ageusia	117 (29)	268 (67)
Bodily pain	80 (20)	305 (76)
Nausea	18 (4)	369 (92)
Numbness	6 (1)	379 (94)
Anosmia	130 (32)	253 (63)
Palpitations	51 (13)	332 (82)
Skin disorders	16 (4)	382 (95)
Sleep disturbance	66 (16)	328 (81)
Stomach ache	10 (2)	386 (96)

Supplementary Figures

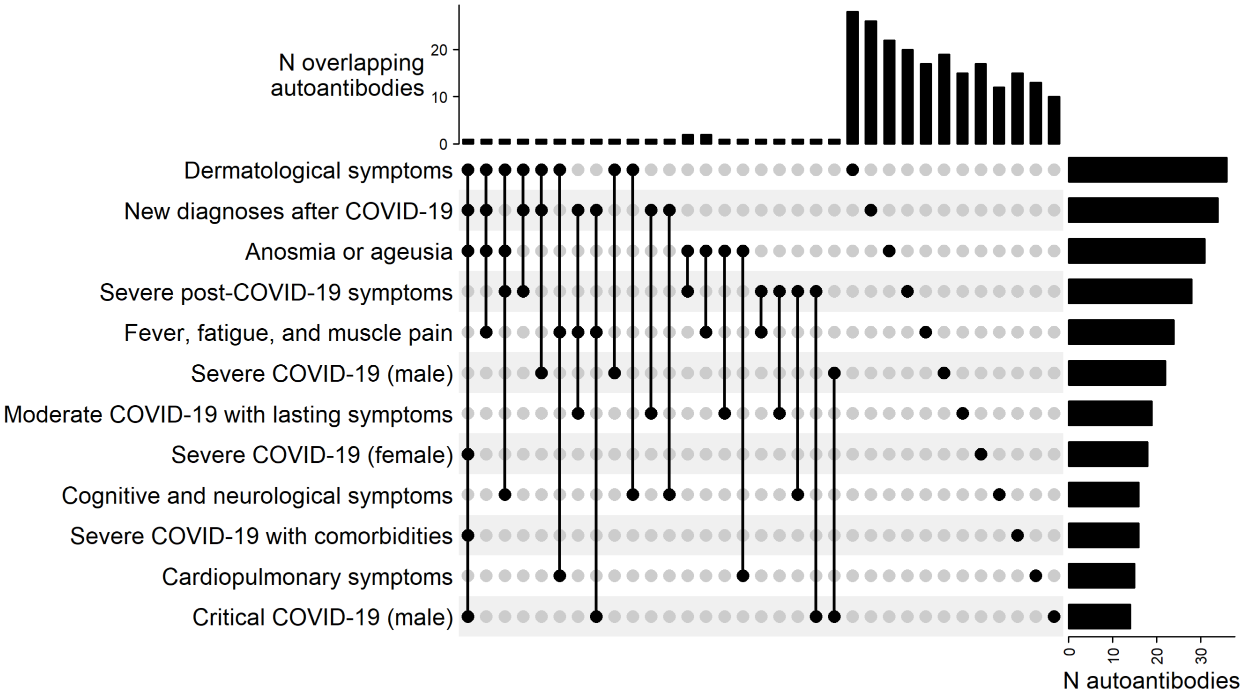

Supplementary Figure 1 | Planar arrays reveal heterogeneous autoantibody profiles in groups of HCW and hospitalized patients.
Row bars depict the number of detected autoantibodies in each group of HCW or hospitalized patients. Interconnected points in the intersection matrix indicate set intersections, i.e., groups sharing one or more detected autoantibodies. Column bars depict the autoantibody profile intersection size, i.e., the number of autoantibodies present in single or multiple groups in accordance with the intersection matrix.

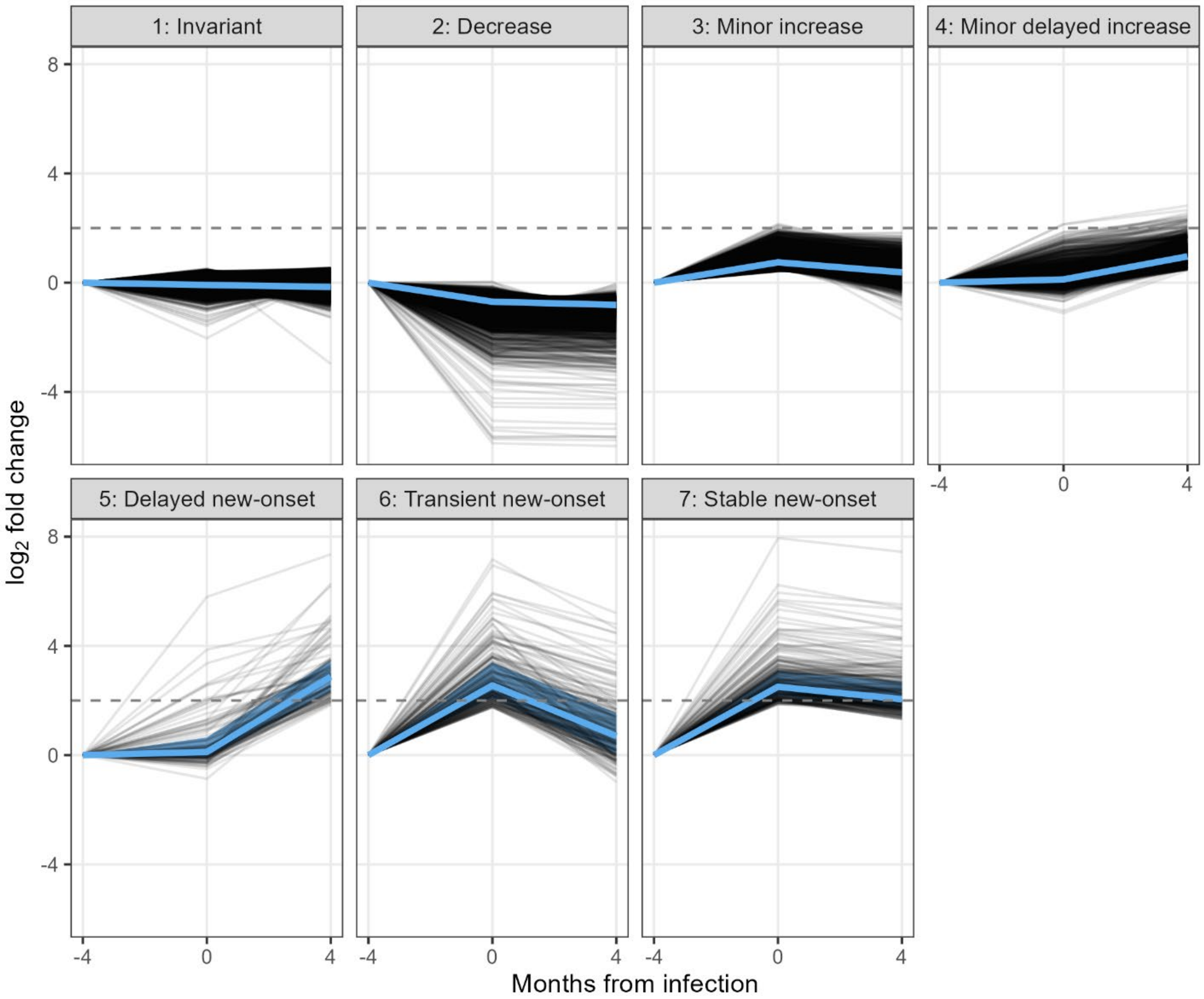

Supplementary Figure 2 | PAM clustering stratifies autoantibody dynamics. PAM clustering with the custom cosine × euclidean distance metric revealed 7 clusters of autoantibody dynamics. Black lines depict autoantibody trajectories (of one autoantibody in one individual). Blue lines and shaded areas represent median and quartiles, respectively.

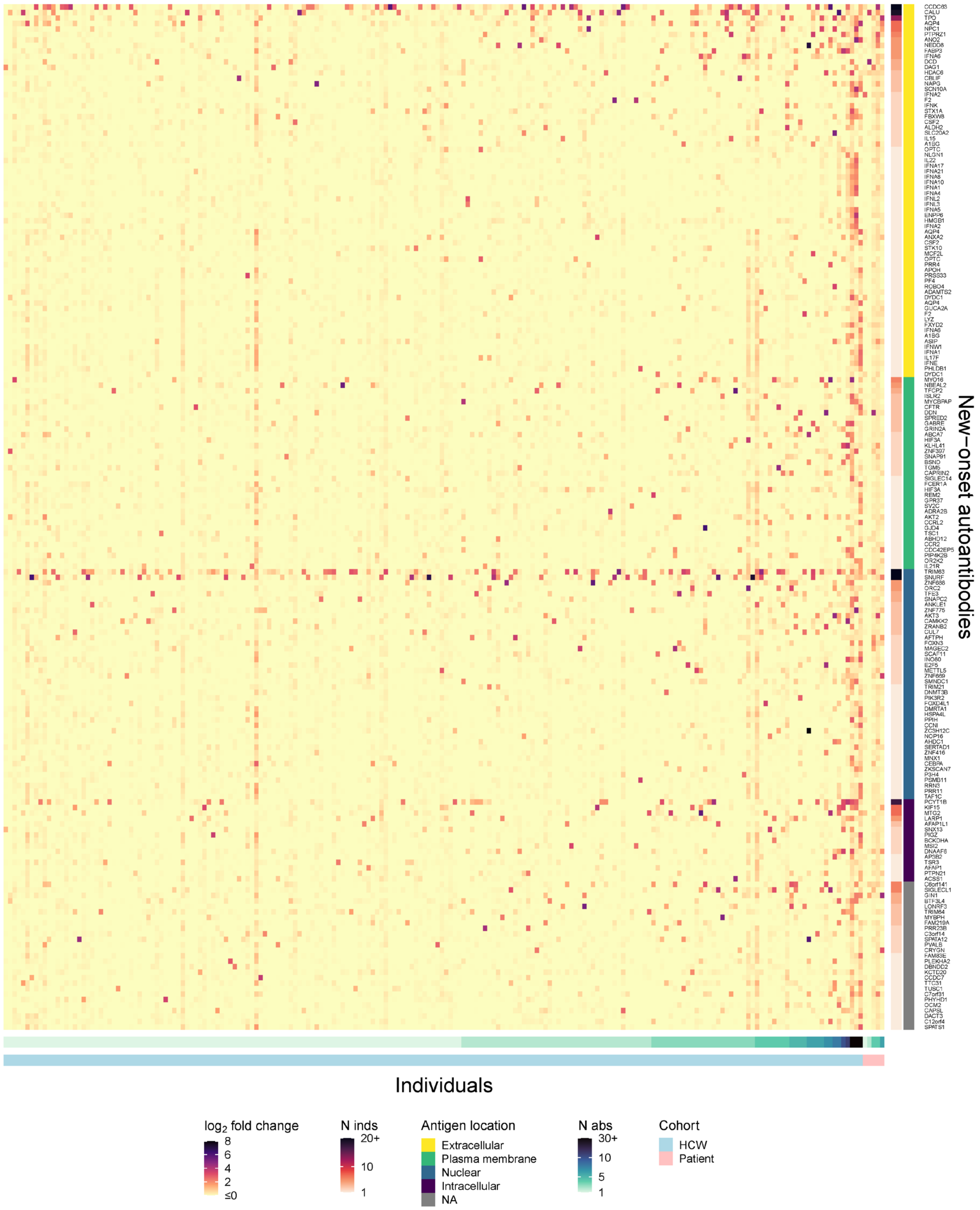

Supplementary Figure 3 | The autoantibody landscape at seroconversion. Heatmap depicting log_2 FC of autoantibody signals at seroconversion. Rows depict detected new-onset autoantibodies arranged by antigen location. Antigens may lack annotation due to the GO subset mapping (see Methods). Columns show unique individuals with seronegative baseline samples and at least one detected new-onset autoantibody. Individuals are arranged by increasing number of new-onset autoantibodies and cohort. Cell color represents log_2 FC of autoantibody signal intensity at infection. Antigen gene names may occur more than once due to multiple protein representations.

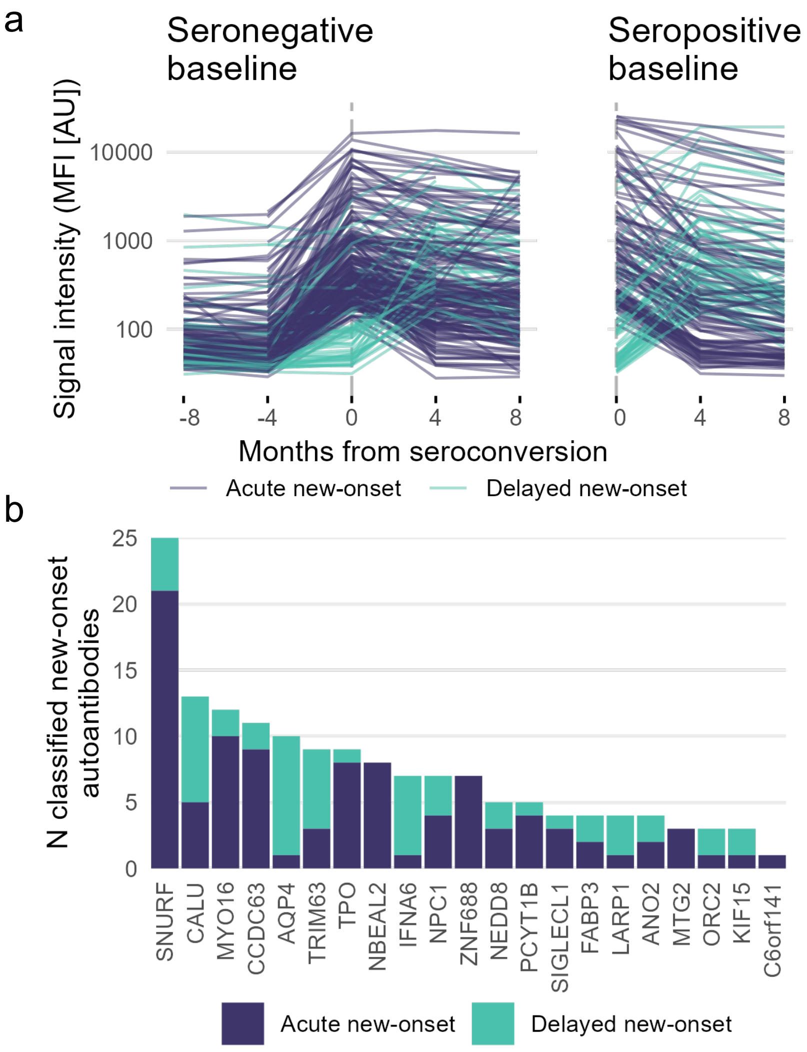

Supplementary Figure 4 | Classification of new-onset autoantibodies in individuals without seronegative baseline sample. **a** Trajectories of new-onset autoantibodies in individuals with seronegative (left) and seropositive (right) baseline sample. **b** Count of new-onset autoantibodies in individuals with seropositive baseline sample.

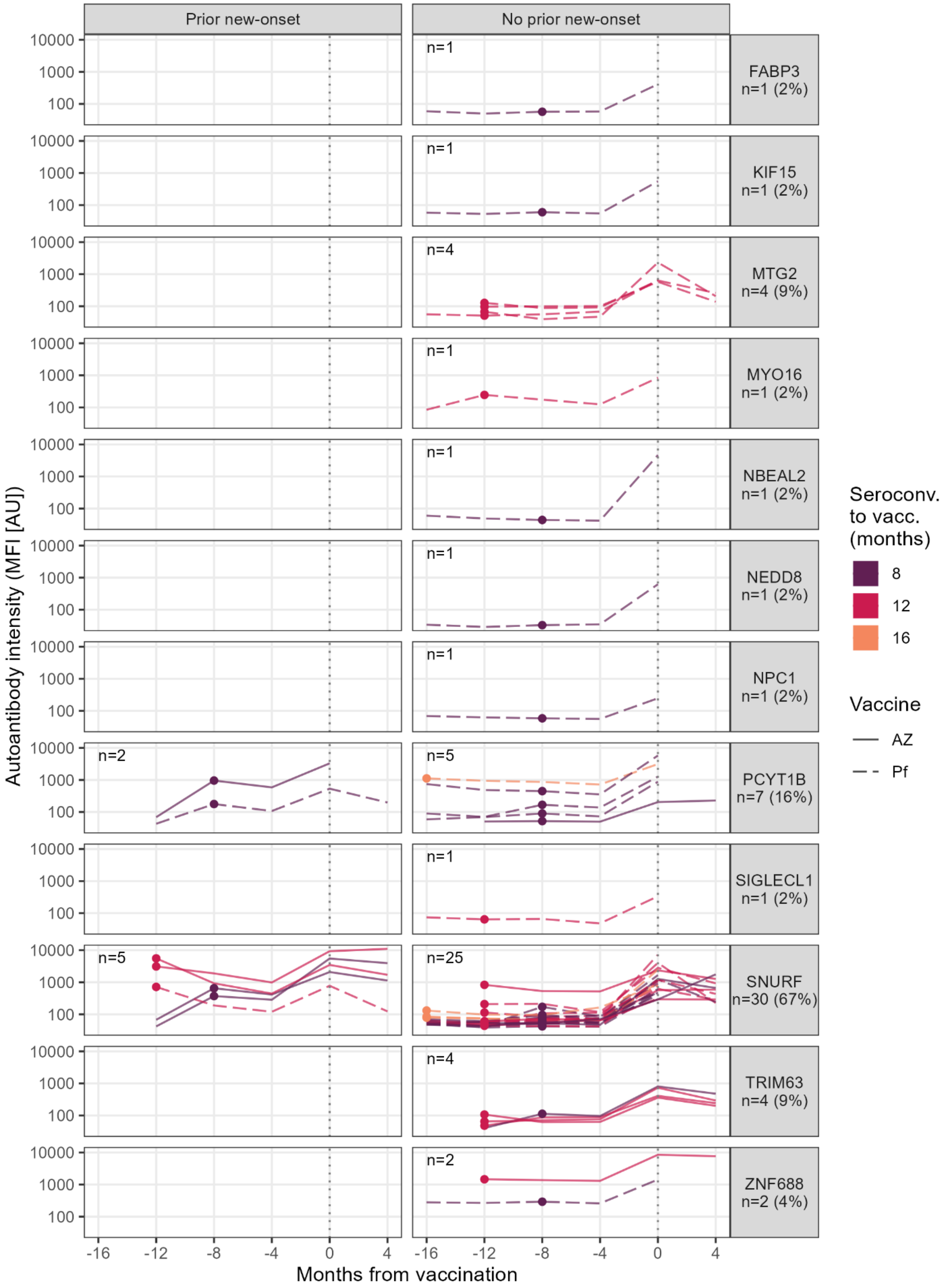

Supplementary Figure 5 | Trajectories of new-onset autoantibodies at vaccination. Lines depict autoantibody trajectories of individuals with a 4-fold increase of any of the 22 prevalent new-onset autoantibodies at vaccination. Points indicate time of seroconversion. Color indicates months between seroconversion and first vaccination. Individuals are separated across panel columns based on any prior new-onset autoantibody towards the corresponding antigen (panel rows) at infection. Percentages were calculated among all HCW with an autoantibody that increased at vaccination (*n*=45).

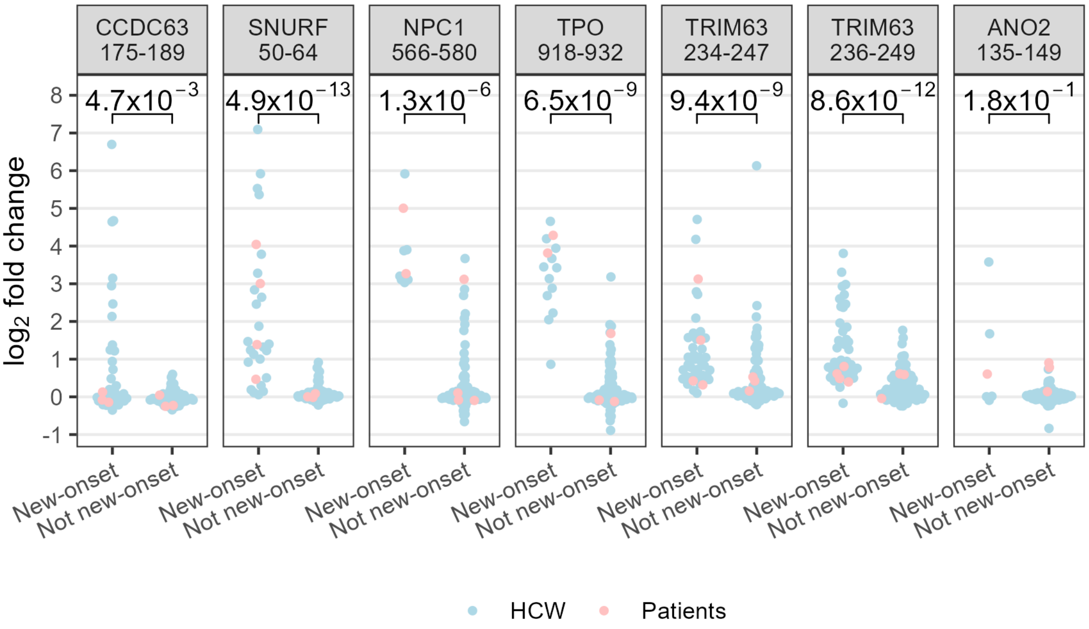

Supplementary Figure 6 | Autoantibodies against the main epitopes in individuals with vs. without the respective new-onset autoantibody. Points correspond to individuals. Categories on the x-axis indicate the prior classification of individuals' autoantibody trajectories towards the protein fragment corresponding to the peptide indicated in the panel titles. Levels on the y-axis correspond to the peptide indicated in the panel titles. Brackets indicate q-values from two-sided Mann-Whitney U tests with Benjamini-Hochberg correction.

a

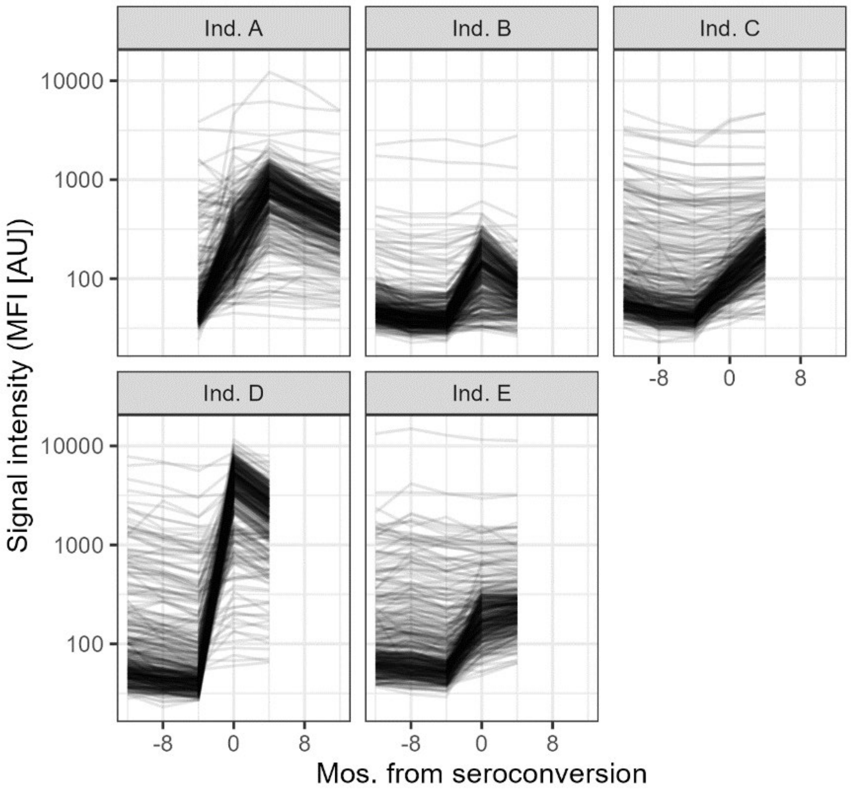

b

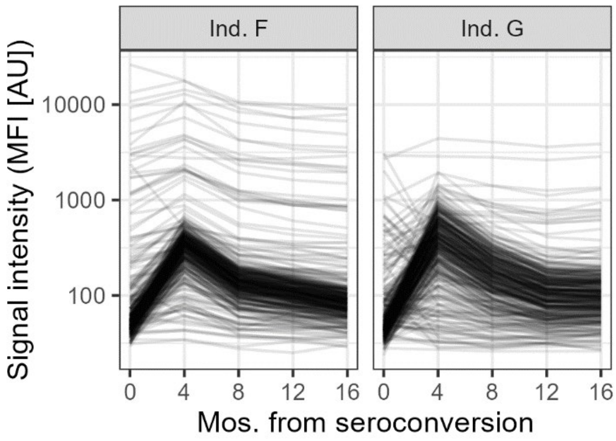

Supplementary Figure 7 | Individuals with deviating global autoantibody patterns. **a** Autoantibody line plots of the 5 individuals with high fold change at seroconversion across most autoantibodies. These individuals were identified using PCA and excluded from PAM clustering. **b** Autoantibody line plots of the 2 individuals with high fold change across most autoantibodies 4 months after seroconversion. These individuals were identified using PCA and excluded from MNL classification.
