# Supplementary material for: Prevalent and persistent new-onset autoantibodies in mild to severe COVID-19
Source: Nat Commun. 2024 Oct 17;15:8941. doi: 10.1038/s41467-024-53356-5 (PMC11484904; doi:10.1038/s41467-024-53356-5)
Supplement: Supplementary file 2 — Description of Additional Supplementary Files [file 41467_2024_53356_MOESM2_ESM.pdf]

Description of Additional Supplementary Files

Supplementary Data 1 | Protein antigens for analysis of the new-onset autoantibody repertoire.

Supplementary Data 2 | Amino acid sequences of the 22 most prevalent new-onset autoantibodies.

Supplementary Data 3 | Peptide antigens for eptitope mapping.
