# Supplementary material for: Prevalent and persistent new-onset autoantibodies in mild to severe COVID-19
Source: Nat Commun. 2024 Oct 17;15:8941. doi: 10.1038/s41467-024-53356-5 (PMC11484904; doi:10.1038/s41467-024-53356-5)
Supplement: Supplementary file 6 — Reporting Summary [file 41467_2024_53356_MOESM6_ESM.pdf]

nature portfolio

Corresponding author(s): August F. Jernbom
Last updated by author(s): Oct 3, 2024

Reporting Summary

Nature Portfolio wishes to improve the reproducibility of the work that we publish. This form provides structure for consistency and transparency in reporting. For further information on Nature Portfolio policies, see our Editorial Policies and the Editorial Policy Checklist.

Statistics

For all statistical analyses, confirm that the following items are present in the figure legend, table legend, main text, or Methods section.

n/a	Confirmed	
☐	☒	The exact sample size (n) for each experimental group/condition, given as a discrete number and unit of measurement
☐	☒	A statement on whether measurements were taken from distinct samples or whether the same sample was measured repeatedly
☐	☒	The statistical test(s) used AND whether they are one- or two-sided<br>*Only common tests should be described solely by name; describe more complex techniques in the Methods section.*
☐	☒	A description of all covariates tested
☐	☒	A description of any assumptions or corrections, such as tests of normality and adjustment for multiple comparisons
☐	☒	A full description of the statistical parameters including central tendency (e.g. means) or other basic estimates (e.g. regression coefficient) AND variation (e.g. standard deviation) or associated estimates of uncertainty (e.g. confidence intervals)
☐	☒	For null hypothesis testing, the test statistic (e.g. F, t, r) with confidence intervals, effect sizes, degrees of freedom and P value noted<br>*Give P values as exact values whenever suitable.*
☒	☐	For Bayesian analysis, information on the choice of priors and Markov chain Monte Carlo settings
☒	☐	For hierarchical and complex designs, identification of the appropriate level for tests and full reporting of outcomes
☐	☒	Estimates of effect sizes (e.g. Cohen's d, Pearson's r), indicating how they were calculated

Our web collection on statistics for biologists contains articles on many of the points above.

Software and code

Policy information about availability of computer code

Data collection	Data were collected using the Luminex xPONENT® 4.3 software and GenePix Pro 5.1.
Data analysis	All data analysis and statistical analysis was performed in RStudio with R 4.1.2 and 4.2.1, and R packages tidyverse, lubridate, rlang, scales, knitr, pander, httr2, readxl, rstatix, proxy, cluster, caret, MLmetrics, MASS, broom, Omixer, ggpubr, ragg, patchwork, cowplot, GGally, ggsignif, ggfortify, ggdist, ggbeeswarm, ggrepel, ComplexHeatmap, and circlize. The abtrac package for antibody trajectory clustering, v0.1.0, implemented in R 4.4.0, is available on GitHub at https://github.com/jernbom/abtrac and archived on Zenodo at doi.org/10.5281/zenodo.13882631.

For manuscripts utilizing custom algorithms or software that are central to the research but not yet described in published literature, software must be made available to editors and reviewers. We strongly encourage code deposition in a community repository (e.g. GitHub). See the Nature Portfolio guidelines for submitting code & software for further information.

Data

Policy information about availability of data

All manuscripts must include a data availability statement. This statement should provide the following information, where applicable:

- Accession codes, unique identifiers, or web links for publicly available datasets
- A description of any restrictions on data availability
- For clinical datasets or third party data, please ensure that the statement adheres to our policy

Source data are provided with this paper. The antibody repertoire data generated in this study have been registered in the SciLifeLab Data Repository under accession code 26318929 [www.doi.org/10.17044/scilifelab.26318929.v1]. The antibody repertoire data are available under restricted access due to data privacy laws. Access can be obtained by researchers who meet the specified criteria by submitting a request through the Data Repository67. The processed antibody repertoire data are provided in the Source Data file. The self-reported post-COVID-19 symptom data are protected and are not available due to data privacy laws. The processed post-COVID-19 symptom data are provided in the Supplementary Information and Source Data file.

Research involving human participants, their data, or biological material

Policy information about studies with human participants or human data. See also policy information about sex, gender (identity/presentation), and sexual orientation and race, ethnicity and racism.

Reporting on sex and gender	Self-reported sex was used in this study. Participants were selected for this study irrespective of sex or gender to maximize sample size. Sex was considered in statistical tests where appropriate. 81% of cohort participants self-reported as female.
Reporting on race, ethnicity, or other socially relevant groupings	No information on social groupings was used in this study.
Population characteristics	The age (mean[SD]) of the HCW, hospitalized COVID-19 patient, neuro-COVID, and pre-pandemic HC cohrts was 45 (11), 57 (13), 62 (16), and 24 (6) years, respectively.
Recruitment	Participants of the COMMUNITY study were selected for study inclusion based on time of seroconversion and amount of self-reported symptoms post-COVID-19. Pre-pandemic healthy controls were selected for study inclusion based on availability of paired pre-pandemic CSF and serum samples. Neuro-COVID participants were included based on a positive PCR for SARS-CoV-2 in upper and/or lower airway samples, and at least one new-onset neurological symptom and presence of anti-SARS-CoV-2 IgG in serum, or typical COVID-19 symptoms in combination with pulmonary ground-glass opacities and consolidations on computed tomography scan of the thorax.
Ethics oversight	The Swedish Ethical Review Authority and the Regional Ethical Review Board in Uppsala, Sweden approved the study protocols.

Note that full information on the approval of the study protocol must also be provided in the manuscript.

Field-specific reporting

Please select the one below that is the best fit for your research. If you are not sure, read the appropriate sections before making your selection.

☒ Life sciences ☐ Behavioural & social sciences ☐ Ecological, evolutionary & environmental sciences

For a reference copy of the document with all sections, see nature.com/documents/nr-reporting-summary-flat.pdf

Life sciences study design

All studies must disclose on these points even when the disclosure is negative.

Sample size	No statistical methods were used to determine sample size. The sample size was based on the number of participants in the COMMUNITY cohort that fulfilled the inclusion criteria described in the Methods. The combination of cohorts and experimental methods is sufficient for the performed analysis.
Data exclusions	Data from individuals with an increase in most autoantibody trajectories at seroconversion were identified using PCA and excluded from further analysis.
Replication	Autoantibody data was captured once for each sample due to restricted availability of sample material in-house.
Randomization	This is an observational study, without any intervention. Group randomization is therefore not relevant to this study.
Blinding	Research technicians were unaware of the participants' characteristics and conditions at the time of data collection.

Reporting for specific materials, systems and methods

We require information from authors about some types of materials, experimental systems and methods used in many studies. Here, indicate whether each material, system or method listed is relevant to your study. If you are not sure if a list item applies to your research, read the appropriate section before selecting a response.

Materials & experimental systems

n/a	Involved in the study	
☐	☒	Antibodies
☒	☐	Eukaryotic cell lines
☒	☐	Palaeontology and archaeology
☒	☐	Animals and other organisms
☒	☐	Clinical data
☒	☐	Dual use research of concern
☒	☐	Plants

Methods

n/a	Involved in the study	
☒	☐	ChIP-seq
☒	☐	Flow cytometry
☒	☐	MRI-based neuroimaging

Antibodies

Antibodies used

Bead arrays:
Secondary antibody: F(ab')2-Goat anti-Human IgG Fc Secondary Antibody, PE, H10104, 2306068, Invitrogen. 0.4 µg/mL.
Primary antibody for positive control: AffiniPure Rabbit Anti-Human IgG (H+L), 309-005-082, 158372, Jackson ImmunoResearch. 17 µg/mL.

Planar arrays:
Secondary antibody: Goat anti-Human IgG (H+L) Cross-Adsorbed Secondary Antibody, Alexa Fluor 647, A21445, Invitrogen/Life Technology. 1:15000.
Spot detection antibody: Hen anti-His6ABP IgY, Immunotech HPA, Stockholm, Sweden. 1:40000.
Secondary antibody: Goat anti-Chicken IgY (H+L) Secondary Antibody, Alexa Fluor 555, A21437, Invitrogen/Life Technology. 1:15000.
Spot detection antibody: Rabbit anti-C-tag, GTX18591, GeneTex. 1:2000.
Secondary antibody: Donkey anti-Rabbit IgG (H+L) Highly Cross-Adsorbed Secondary Antibody, Alexa Fluor 555, A31572, Invitrogen/Life Technology. 1:15000.

Validation

AffiniPure Rabbit Anti-Human IgG (H+L), 309-005-082, 158372, Jackson ImmunoResearch:
Product specifications: Based on immunoelectrophoresis and/or ELISA, the antibody reacts with whole molecule human IgG. It also reacts with the light chains of other human immunoglobulins. No antibody was detected against non-immunoglobulin serum proteins. The antibody has been tested by ELISA and/or solid-phase adsorbed to ensure minimal cross-reaction with mouse serum proteins, but it may cross-react with immunoglobulins from other species
https://www.jacksonimmuno.com/catalog/products/309-005-082

Plants

Seed stocks

Report on the source of all seed stocks or other plant material used. If applicable, state the seed stock centre and catalogue number. If plant specimens were collected from the field, describe the collection location, date and sampling procedures.

Novel plant genotypes

Describe the methods by which all novel plant genotypes were produced. This includes those generated by transgenic approaches, gene editing, chemical/radiation-based mutagenesis and hybridization. For transgenic lines, describe the transformation method, the number of independent lines analyzed and the generation upon which experiments were performed. For gene-edited lines, describe the editor used, the endogenous sequence targeted for editing, the targeting guide RNA sequence (if applicable) and how the editor was applied.

Authentication

Describe any authentication procedures for each seed stock used or novel genotype generated. Describe any experiments used to assess the effect of a mutation and, where applicable, how potential secondary effects (e.g. second site T-DNA insertions, mosiacism, off-target gene editing) were examined.
