# Supplementary material for: Prevalent and persistent new-onset autoantibodies in mild to severe COVID-19
Source: Nat Commun. 2024 Oct 17;15:8941. doi: 10.1038/s41467-024-53356-5 (PMC11484904; doi:10.1038/s41467-024-53356-5)
Supplement: Supplementary file 7 — Source Data [file 41467_2024_53356_MOESM7_ESM.pdf]

nature portfolio

Peer Review File

Prevalent and persistent new-onset autoantibodies in mild to severe COVID-19

Corresponding Author: Dr August Jernbom

This file contains all reviewer reports in order by version, followed by all author rebuttals in order by version.

Version 0:

Reviewer comments:

Reviewer #1

(Remarks to the Author)
The paper describes through epitope mapping that 26 main epitopes of selected new-onset autoantibodies emerge in patients with COVID-19 and that these autoantibodies may have sequence similarities indicative of molecular mimicry with the conserved fusion peptide of the SARS-CoV-2 Spike glycoprotein. It is very intriguing that several antibodies directed towards molecules involved in muscular function, or present in the heart and brain, could be involved in the pathogenesis of post-COVID sequelae. They also identified an association between some of these autoantibodies and the severity of neuro-psychiatric symptoms. The presence of autoantibodies was also found in the CSF of patients, suggesting an impairment of the BBB in this subset.
Major comments.
The main limit of this study, as stated also by the authors, is the collection of patients who self-reported a variety of symptoms that can be referred to post-COVID syndrome (long-COVID) but that were not assessed by a physician. Moreover, the authors aim at addressing whether these autoantibodies were associate with clinical manifestations, however, the sample size seems quite small to be able to depict this data.
In the initial phase of the investigation the authors identified subsets of patients with post-COVID-19 symptoms in order to pool sera. Among the groups two are defined as "multiple and severe symptoms" and "multiple symptoms after severe COVID-19". The difference between them is not at al clear. Additionally, a detailed definition of patient features employed to classify them into each group is lacking.
The authors had access to CSF from healthy subjects. Its origin needs to be clarified. Why was lumbar puncture performed in this cohort?
Because CSF from COVID-19 patient was collected when clinically indicated, this may introduce a significant selection bias. Although it is unreasonable to ask for CSF samples from COVID-19 patients with no indication to perform the procedure, it would still be fundamental to provide some clinical data comparing the cohort of COVID-19 patients who underwent LP and those who did not, especially in terms of neurological manifestations.
Anti TPO is an interesting antibody since shows homologies with the Spike glycoprotein subunit 1. However, the prevalence of this antibody seems not relevant for clinical purposes (4%), and the authors stress that anti-NPC1 IgG were found alone in 4 individuals, which is a small number that would require verification in larger cohorts.
Among all the patients, it would be of interest to know how many suffered already from symptoms of post-COVID including migraine, headache, fibromyalgia already before inclusion.

Minor
Did any of the patients suffered from myositis? Any evidence of an association between autoantibodies and CPK/LDH myoglobin levels?
Did the authors tested other “classical” autoantibodies in this cohort including antinuclear and anti-ENA?
SARS-CoV-2 virus has significantly changed over time. any evidence that different virus variants could have differently impacted on ab emergence?
Could the authors observe any correlation between the epidemiological curve and the onset of autoabs?
Table 1 is not clear. Number of patients and cohort in which autoabs were studied should be added.
Do any of these autoabds have a potential usage as a diagnostic biomarker for post-COVID?
Figure 4 is difficult to understand and the message it wants to convey is not clear.

Reviewer #2

(Remarks to the Author)
The work presents a wide and detailed analysis of autoantibodies generated during COVID-19 and 16 months after. The analysis of the IgG autoantibody response over time, the autoantibody specificity and the contribution of vaccination to this responses present new, important aspects of the autoimmune response generated in response to COVID-19.
The work is significant to the field of COVID-19 and to autoimmunity in general, since it is showing that new autoantibody prevalence is related to disease severity and that new-onset autoimmune responses in patients persist for more than 1 year after infection.
The experiments presented largely support the conclusions.

However, the manuscript is missing a detailed comparison with previously published work to put in context the new findings. Specific observations, such as frequency of patient autoreactivity, common antigens involved in molecular mimicry or kinetics of autoreactivity should be compared to other COVID-19 studies.
The frequency of autoimmune reactivity in patients should also be compared. In this study, taken together all the samples analyzed they found IgG reactivity to 215 protein fragments out of 42,000 in the proteomics library (0.5%). The authors should discuss whether this is similar to the proportion observed in other COVID-19 studies.

On the specific issue of molecular mimicry, a comparison of the antigens identified here to the previously identified ones for COVID-19 in the literature would be very helpful to put the new findings in context. A table including antigen names and references may be way to summarize these data.

Some examples of related literature not cited in the manuscript:
• "Distinguishing features of long COVID identified through immune profiling" PMID:37748514
• "Severe COVID-19 patients exhibit elevated levels of autoantibodies targeting cardiolipin and platelet glycoprotein with age: a systems biology approach" PMID: 37620330

Minor points:
There is a lack of detail in the explanations of different methods. For example, which criteria was used to determine whether reactivity for a particular protein fragment or full-length protein was considered positive or negative.

line 49: "autoantibodies and their connection to the course of COVID-19"
line 264: "three new-onset autoantibodies connected to increased severity of neuropsychiatric symptoms post-COVID-19"
line 289:"indications of connections to muscular symptoms"
The autoantibodies are 'associated', not directly 'connected' to disease or symptoms.

Fig. S1. please expand the figure legend to explain what do the gray and black circles represent and the lines connecting the circles. It is difficult to understand.

Reviewer #3

(Remarks to the Author)
I co-reviewed this manuscript with one of the reviewers who provided the listed reports. This is part of the Nature Communications initiative to facilitate training in peer review and to provide appropriate recognition for Early Career Researchers who co-review manuscripts.

Reviewer #4

(Remarks to the Author)
This is an elegant and timely manuscript that addresses a very important question in the field of acute infections, and SARS-CoV2 infection in particular. Does COVID generate new autoantibodies and new onset autoimmune diseases? While the latter question can only be addressed with large epidemiology studies, the former question is addressed here. Several studies have demonstrated new onset autoantibodies in COVID-19 and more recently in pre-pandemic infected ICU patients. In those studies, samples were compared at the time of infection as the earliest time point. Up until now, no studies, to this reviewer's knowledge, has compared pre-pandemic samples, or in this case pandemic samples, in which a sample could be directly compared prior to infection with a sample collected post infection. The authors take advantage of such a cohort in which samples were collected not just prior to infection and soon after infection, but at multiple time points over many months. They use large-scale protein fragment arrays to identify candidate antigens using a smaller discovery cohort, then focus those antigens onto Luminex bead arrays to study much larger numbers of samples, a method they have pioneered. For example, they have used this approach previously, including to discover anoctamin as a candidate MS autoantigen linked to EBV.

Using this approach, here they discover new candidate autoantigens following acute SARS-CoV2 infection showing high prevalence that correlates positively with disease severity. Some autoantibodies are transient, others are persistent, and still others appear with delayed onset. They go on to also compare paired blood and CSF samples. These results will serve as foundational studies for future studies on infectious triggers of autoantibodies, vaccines and vaccine safety, tolerance, molecular mimicry and other molecular mechanisms, and long COVID.

Specific concerns and suggestions are below.

1. Although not required, it would be useful to know if the interferon autoantibodies have receptor blocking activity, and if so if this is found in the pre-infection sample or if it evolved after the infection through the affinity maturation process. Several labs have established this assay so perhaps in future studies these could be tested.

2. Did the authors look at other classes of antibody, for example IgM or IgA? IgM responses in particular would suggest this is truly a new-onset autoantibody as opposed to a recall response of a low level Ig at baseline. This could be easily done for a few antigens and would seem most relevant for anti-interferons, but again not necessary.

3. The authors should review and consider adding this reference (Shaw et al, PMC8689695). This paper suggests that IFN autoantibodies may be transient (and probably inducible) in COVID-19 but not in APS1.

4. A limitation of the study is the use of protein fragments rather than full-length proteins. Did the authors attempt to validate some of these results using full length proteins? For the interferon part, they comprehensively did this, using fragments, commercially available proteins, etc. It would be useful to describe any validation they did. The easiest to validate would be TPO. If validated using full length protein, then this suggests future studies could be done using electronic health records, and would facilitate validation by other groups who do not have protein fragments available. This reviewer recognizes that the assays used here are research-grade, so a comprehensive validation effort is not indicated here.

5. Did the authors do any other homology comparisons between other SARS-CoV2 proteins and their newly-identified autoantigens? Nucleocapsid in particular has been shown to be a molecular mimic in MIS-C (medRxiv 2023.05.26.23290373).

6. Did any of the patients who developed new-onset aquaporin-4 autoantibodies develop CNS disease?

7. The authors should consider also noting new-onset autoantibodies have been observed in patients with acute influenza, and association with anti-interferons and other cytokines (Feng, 36752204).

8. The vaccine data are interesting and important; however, it may be prudent to carefully characterize the interpretation as not supporting induction of autoantibodies or autoimmunity by this or other vaccines. There exists a strong anti-vaccine group of people who could seize on this finding and use it to show potential harm from vaccination. The authors should reiterate these results do not support this view, and moreover that the candidate antigens have not been linked to autoimmunity, long COVID, or vaccination.

9. The authors should also supplement reference 15 by adding references to at least 2 papers showing absence of new or increased autoantibodies in vaccine trials (Arunachalam, Nature, PMID 34252919; and Sarin, JCI Insight, PMID 38456511). General absence of new autoantibodies in response to vaccines was also observed in a large cohort of patients with SLE and other autoimmune diseases (in press in JCI Insight).

10. In the discussion, which is very well-written and balanced, the authors speculate regarding molecular mimicry. This is very reasonable and is supported by their findings. However, this reviewer recommends softening the language. For example instead of saying "indicative of molecular mimicry," they could say suggestive of, or consistent with a mechanism invoking molecular mimicry. This relates back to the vaccine link since the epitope is found both in the vaccine and the natural virus. The authors could also consider instead describing their findings as "cross-reactive" epitopes since target antigens are intracellular and it is unclear how this would drive a break in tolerance. This is somewhat semantics but nevertheless worth considering.

11. Several very large epidemiology studies have emerged showing an increased incidence of autoimmunity, including thyroid autoimmunity. The authors should consider linking their findings to these large epi studies. Examples include Tesch (PMID 37335408), Lim (PMID 37801317) and others.

Version 1:

Reviewer comments:

Reviewer #1

(Remarks to the Author)
The authors properly responded to all raised comments.

Reviewer #2

(Remarks to the Author)

Reviewer #3

(Remarks to the Author)
I co-reviewed this manuscript with one of the reviewers who provided the listed reports. This is part of the Nature

Communications initiative to facilitate training in peer review and to provide appropriate recognition for Early Career Researchers who co-review manuscripts.

Reviewer #4

(Remarks to the Author)
I have reviewed the revised manuscript as well as the critiques and rebuttal to all other reviewers. My initial review was very positive and most of my concerns have been addressed. The authors should look at some of the references that were suggested to be added since (I think) several that had been in MedRxiv are now published in peer-reviewed journals (eg Bodansky paper in Nature, the lupus vaccine paper in JCI Insight.

A concern remains regarding validation using whole antigens. It would not be at all difficult to pick a few key antigens that are clinically relevant such as thyroperoxidase and aquaporin-4 and validate the autoantibody results on an orthogonal platform such as ELISA, bead-based or other methods. Antigens are readily available and this group has pioneered micro-bead based AAb profiling. Two outcomes are possible, they either correlate or they don't. If they do, then other groups can validate on other cohorts. If not, then they can describe why there is a discrepancy.

Open Access This Peer Review File is licensed under a Creative Commons Attribution 4.0 International License, which permits use, sharing, adaptation, distribution and reproduction in any medium or format, as long as you give appropriate credit to the original author(s) and the source, provide a link to the Creative Commons license, and indicate if changes were made.
In cases where reviewers are anonymous, credit should be given to 'Anonymous Referee' and the source.
The images or other third party material in this Peer Review File are included in the article’s Creative Commons license, unless indicated otherwise in a credit line to the material. If material is not included in the article’s Creative Commons license and your intended use is not permitted by statutory regulation or exceeds the permitted use, you will need to obtain permission directly from the copyright holder.
To view a copy of this license, visit https://creativecommons.org/licenses/by/4.0/

REVIEWER COMMENTS

Reviewer #1 (Remarks to the Author):

The paper describes through epitope mapping that 26 main epitopes of selected new-onset autoantibodies emerge in patients with COVID-19 and that these autoantibodies may have sequence similarities indicative of molecular mimicry with the conserved fusion peptide of the SARS-CoV-2 Spike glycoprotein. It is very intriguing that several antibodies directed towards molecules involved in muscular function, or present in the heart and brain, could be involved in the pathogenesis of post-COVID sequelae. They also identified an association between some of these autoantibodies and the severity of neuro-psychiatric symptoms. The presence of autoantibodies was also found in the CSF of patients, suggesting an impairment of the BBB in this subset.

Authors' reply: We thank Reviewer 1 for their insightful assessment and valuable comments. We have thoroughly considered all specific points raised below and addressed them in full, either through adjustments in the Manuscript and Supplementary material, or by highlighting existing parts of the Manuscript and Supplementary material relevant to the point raised. We feel that their valuable feedback has considerably improved the manuscript.

Major comments.

The main limit of this study, as stated also by the authors, is the collection of patients who self-reported a variety of symptoms that can be referred to post-COVID syndrome (long-COVID) but that were not assessed by a physician. Moreover, the authors aim at addressing whether these autoantibodies were associate with clinical manifestations, however, the sample size seems quite small to be able to depict this data.

Authors' reply: We agree that the sample size and symptom prevalence of our study is limiting the power of achieving our secondary study goal of detecting associations of new-onset autoantibodies with post-COVID-19 symptoms. Throughout the manuscript, we have consistently avoided reporting lack of detected association as evidence of no association. We have adjusted the Discussion paragraph on study limitations highlight the importance of the limited symptom prevalence.

In the initial phase of the investigation the authors identified subsets of patients with post-COVID-19 symptoms in order to pool sera. Among the groups two are defined as "multiple and severe symptoms" and "multiple symptoms after severe COVID-19". The difference between them is not at al clear. Additionally, a detailed definition of patient features employed to classify them into each group is lacking.

Authors' reply: We have added Table S2, detailing the information of participant features employed to classify them into each group, including the two groups mentioned above.

The authors had access to CSF from healthy subjects. Its origin needs to be clarified. Why was lumbar puncture performed in this cohort?

Authors' reply: The CSF samples from healthy subjects were collected within the Uppsala Psychiatric Patients Samples (UPP) cohort for the purpose of serving as healthy control samples. Ethical permission for their collection and use was obtained. We have clarified this in the cohort description in Methods.

Because CSF from COVID-19 patient was collected when clinically indicated, this may introduce a significant selection bias. Although it is unreasonable to ask for CSF samples from COVID-19 patients with no indication to perform the procedure, it would still be fundamental to provide some clinical data comparing the cohort of COVID-19 patients who underwent LP and those who did not, especially in terms of neurological manifestations.

Authors' reply: We agree that this data is of importance. We have considerably updated the demographics table (Table S1) with data on acute disease severity, neurological manifestations, and preexisting comorbidities. In line with these data, we consider the discovery cohort of COVID-19 patients and the validation cohort of neuro-COVID patients comparable. The major clinical difference between the cohorts is the presentation of new-onset neurological manifestations on infection. This difference is necessary as lumbar puncture was only performed when clinically indicated, as noted by the reviewer. In line with this, we limit our interpretation of the detected new-onset autoantibodies in CSF to stating that they are detectible in both CSF and blood, which suggests that they can pass the blood-brain barrier, as stated in the Discussion (in the paragraph on anti-SNURF).

Anti TPO is an interesting antibody since shows homologies with the Spike glycoprotein subunit 1. However, the prevalence of this antibody seems not relevant for clinical purposes (4%), and the authors stress that anti-NPC1 IgG were found alone in 4 individuals, which is a small number that would require verification in larger cohorts.

Authors' reply: We agree that the co-occurrence and tentative link between anti-Spike, anti-NPC1, and anti-TPO IgG is interesting, and we agree it warrants further study and verification in larger cohorts, as raised in the Discussion. We agree that the clinical relevance of the anti-TPO antibodies that we detect remains uncertain at this stage, but we do believe that it warrants attention due to its relationship with antithyroid diseases and the reports of new-onset antithyroid disease after COVID-19, as we elaborate in the Discussion.

Among all the patients, it would be of interest to know how many suffered already from symptoms of post-COVID including migraine, headache, fibromyalgia already before inclusion.

Authors' reply: While we agree that this information would be of interest, collection of this data was not considered in the early pandemic due to incomplete knowledge of the coming emergence of post-COVID-19 symptoms. Our study necessitates the use of samples collected within weeks of the outbreak of COVID-19 to follow the emergence of new-onset autoantibodies after naïve infection. We have adjusted the Discussion paragraph on study limitations to reflect this.

Minor

Did any of the patients suffered from myositis? Any evidence of an association between autoantibodies and CPK/LDH myoglobin levels?

Authors' reply: We have no information that any of the hospitalized COVID-19 patients suffered from myositis. We do not have access to measures of CPK/LDH myoglobin levels in the cohorts.

Did the authors tested other "classical" autoantibodies in this cohort including antinuclear and anti-ENA?

Authors' reply: We exclusively tested autoantibodies using the methods mentioned in the manuscript. Our large planar arrays contain protein fragments representing known autoantigens, e.g., SSA/Ro, Ro52/TRIM21, SSB/La, Scl70/TOP1, Jo1, histones, and CENPA-C. In this study, autoantibodies against these antigens were not notable in the antigen selection and/or new-onset stages of analysis. Some of the detected autoantibodies, such as anti-SNURF, are targeting nuclear proteins.

SARS-CoV-2 virus has significantly changed over time. any evidence that different virus variants could have differently impacted on ab emergence?

Authors' reply: While our study was not set up to detect such associations, we agree that this is an interesting line of study for future work.

Could the authors observe any correlation between the epidemiological curve and the onset of autoabs?

Authors' reply: Although this is difficult to accurately estimate and not sufficiently evidenced for reporting, the incidence of new-onset autoantibodies was approximately proportional to the incidence of seroconversion.

Table 1 is not clear. Number of patients and cohort in which autoabs were studied should be added.

Authors' reply: We have adjusted Table 1 to make it clear that the autoantibodies indicated were studied in the HCW and hospitalized COVID-19 patients, and provided the cohort size.

Do any of these autoabds have a potential usage as a diagnostic biomarker for post-COVID?

Authors' reply: We agree that autoantibody biomarkers for post-COVID-19 is an interesting prospect and a valuable line of future research. Our study relies on longitudinal measurements of autoantibodies, which provides an opportunity to study the immune system across infection. However, longitudinal measurements relative to a pre-infectious baseline are not suited to clinical use due to the need for prospective testing. We encourage future work building upon this and other studies for the development of diagnostic autoantibodies for post-COVID-19.

Figure 4 is difficult to understand and the message it wants to convey is not clear.

Authors' reply: We have clarified the description of Figure 4 and the corresponding paragraph in the Results.

Reviewer #2 (Remarks to the Author):

The work presents a wide and detailed analysis of autoantibodies generated during COVID-19 and 16 months after. The analysis of the IgG autoantibody response over time, the autoantibody specificity and the contribution of vaccination to this responses present new, important aspects of the autoimmune response generated in response to COVID-19.

The work is significant to the field of COVID-19 and to autoimmunity in general, since it is showing that new autoantibody prevalence is related to disease severity and that new-onset autoimmune responses in patients persist for more than 1 year after infection.

The experiments presented largely support the conclusions.

Authors' reply: We thank Reviewer #2 for their thoughtful and thorough review of our manuscript. We greatly appreciate the referee's recognition of the significance of our work in understanding the long-term autoantibody response to COVID-19 and its implications for both COVID-19 and autoimmunity research. We are grateful for their positive assessment of our experiments and conclusions.

However, the manuscript is missing a detailed comparison with previously published work to put in context the new findings. Specific observations, such as frequency of patient autoreactivity, common antigens involved in molecular mimicry or kinetics of autoreactivity should be compared to other COVID-19 studies.

Authors' reply: We appreciate the referee's suggestion regarding the comparison with the literature. However, our manuscript aims to present novel findings and insights, and while we acknowledge the importance of context, we focused on delivering new data and observations. A detailed comparison with previously published work would be highly appropriate in a dedicated review article, which aims to synthesize and critically analyze existing knowledge in the field. We do, however, recognize the importance of contextualizing our work with several new key references, including those indicated by Reviewer #2 below. To this end, we have added these and several other additional references to the manuscript.

The frequency of autoimmune reactivity in patients should also be compared. In this study, taken together all the samples analyzed they found IgG reactivity to 215 protein fragments out of 42,000 in the proteomics library (0.5%). The authors should discuss whether this is similar to the proportion observed in other COVID-19 studies.

Authors' reply: While we agree that a detailed comparison of observed autoantibody frequencies is an interesting question, we acknowledge that this is a major undertaking requiring careful consideration and planning which requires a dedicated systematic review. There are several major technical challenges that need to be addressed to make a robust and informative comparison of autoantibody frequencies.

1) Different autoantibody screening methods utilize different antigen collections and antigen representations (varying by protein/fragment/peptide length, proteome coverage, post-translational modifications, etc.), thus representing different sets of antigenic space. Therefore, comparison of reactivity frequencies needs careful consideration, including degree of array overlap on the amino acid level.

2) Different autoantibody screening studies have different aims for different parts of the analysis. For instance, our studies utilize a two-step approach where we perform untargeted screening on planar arrays and targeted screening on bead-based arrays. As the former represents an exploratory discovery stage, we apply reactivity cutoffs tailored to a broad selection of antigens. In contrast, the latter stage forms the basis of our continued analysis and requires a much more stringent reactivity cutoff.

With these points in mind, a comparison of autoantibody reactivity frequencies in any less than a well-made systematic review would produce inaccurate findings and would not be informative for the field. The referee’s suggestion is therefore not within the scope of our study. We would highly welcome community initiatives of systematic reviews to address this question.

On the specific issue of molecular mimicry, a comparison of the antigens identified here to the previously identified ones for COVID-19 in the literature would be very helpful to put the new findings in context. A table including antigen names and references may be way to summarize these data.

Authors’ reply: We fully agree that a thorough collection of the antigens previously identified in the context of COVID-19 would be of great value for the field and of interest for contextualization of our findings. However, there is an extensive record of such literature (currently 270 results in PubMed for ((COVID-19) OR (SARS-CoV-2)) AND ("molecular mimicry"), and their collection requires a dedicated systematic review. In our manuscript we do, however, comment that our findings represent the first records indicative of molecular mimicry with TRIM63 and CCDC63. We agree that this should also be detailed for NPC1, and have adjusted the paragraph on NPC1 in the Discussion accordingly.

Some examples of related literature not cited in the manuscript:

• “Distinguishing features of long COVID identified through immune profiling” PMID:37748514

• “Severe COVID-19 patients exhibit elevated levels of autoantibodies targeting cardiolipin and platelet glycoprotein with age: a systems biology approach” PMID: 37620330

Authors’ reply: As noted above, we have now implemented these references in the manuscript.

Minor points:

There is a lack of detail in the explanations of different methods. For example, which criteria was used to determine whether reactivity for a particular protein fragment or full-length protein was considered positive or negative.

Authors’ reply: The criteria for determining reactivity or new-onset are described in Methods, subsections “Analysis of planar array data”, “Classification of new-onset autoantibodies in individuals with a seronegative baseline sample”, and “Classification of new-onset autoantibodies in individuals without any seronegative baseline sample”.

line 49: "autoantibodies and their connection to the course of COVID-19"

line 264: "three new-onset autoantibodies connected to increased severity of neuropsychiatric symptoms post-COVID-19"

line 289:"indications of connections to muscular symptoms"

The autoantibodies are 'associated', not directly 'connected' to disease or symptoms.

Authors' reply: We agree and have adjusted the manuscript accordingly.

Fig. S1. please expand the figure legend to explain what do the gray and black circles represent and the lines connecting the circles. It is difficult to understand.

Authors' reply: We have clarified the figure legend.

Reviewer #3 (Remarks to the Author):

I co-reviewed this manuscript with one of the reviewers who provided the listed reports. This is part of the Nature Communications initiative to facilitate training in peer review and to provide appropriate recognition for Early Career Researchers who co-review manuscripts.

Authors' reply: We thank Reviewer #3 for their contribution through co-review.

Reviewer #4 (Remarks to the Author):

This is an elegant and timely manuscript that addresses a very important question in the field of acute infections, and SARS-CoV2 infection in particular. Does COVID generate new autoantibodies and new onset autoimmune diseases? While the latter question can only be addressed with large epidemiology studies, the former question is addressed here. Several studies have demonstrated new onset autoantibodies in COVID-19 and more recently in pre-pandemic infected ICU patients. In those studies, samples were compared at the time of infection as the earliest time point. Up until now, no studies, to this reviewer's knowledge, has compared pre-pandemic samples, or in this case pandemic samples, in which a sample could be directly compared prior to infection with a sample collected post infection. The authors take advantage of such a cohort in which samples were collected not just prior to infection and soon after infection, but at multiple time points over many months. They use large-scale protein fragment arrays to identify candidate antigens using a smaller discovery cohort, then focus those antigens onto Luminex bead arrays to study much larger numbers of samples, a method they have pioneered. For example, they have used this approach previously, including to discover anoctamin as a candidate MS autoantigen linked to EBV.

Using this approach, here they discover new candidate autoantigens following acute SARS-CoV2 infection showing high prevalence that correlates positively with disease severity. Some autoantibodies are transient, others are persistent, and still others appear with delayed onset. They go on to also compare paired blood and CSF samples. These results will serve as foundational studies for future studies on infectious triggers of autoantibodies, vaccines and vaccine safety, tolerance, molecular mimicry and other molecular mechanisms, and long COVID.

Authors’ reply: We thank Reviewer #4 for their accurate and comprehensive summary of our work. We greatly appreciate the knowledgeable and helpful specific comments provided below. We have addressed these in full and believe that this has contributed to improving our manuscript.

Specific concerns and suggestions are below.

1. Although not required, it would be useful to know if the interferon autoantibodies have receptor blocking activity, and if so if this is found in the pre-infection sample or if it evolved after the infection through the affinity maturation process. Several labs have established this assay so perhaps in future studies these could be tested.

Authors’ reply: We agree that this knowledge would be highly interesting and can form the basis of a useful follow-up study.

2. Did the authors look at other classes of antibody, for example IgM or IgA? IgM responses in particular would suggest this is truly a new-onset autoantibody as opposed to a recall response of a low level Ig at baseline. This could be easily done for a few antigens and would seem most relevant for anti-interferons, but again not necessary.

Authors’ reply: We agree that a study of new-onset IgM antibodies would be very informative. However, we did not investigate IgM in the present study as we sought to investigate the longitudinal antibody response over several months, which may be incompatible with the transient nature of the IgM response. Studies aiming to replicate our results should assess both IgG and IgM, which is much easier in the context of a smaller set of antigens, as the referee correctly suggests.

3. The authors should review and consider adding this reference (Shaw et al, PMC8689695). This paper suggests that IFN autoantibodies may be transient (and probably inducible) in COVID-19 but not in APS1.

Authors’ reply: We appreciate this suggestion and agree. We have added this reference.

4. A limitation of the study is the use of protein fragments rather than full-length proteins. Did the authors attempt to validate some of these results using full length proteins? For the interferon part, they comprehensively did this, using fragments, commercially available proteins, etc. It would be useful to describe any validation they did. The easiest to validate would be TPO. If validated using full length protein, then this suggests future studies could be done using electronic health records, and would facilitate validation by other groups who do not have protein fragments available. This reviewer recognizes that the assays used here are research-grade, so a comprehensive validation effort is not indicated here.

Authors’ reply: We did not attempt to validate any of the results using full-length proteins. Instead, we validated the results using short peptides as this enabled epitope mapping and evaluation of possible molecular mimicry. The referee’s suggestion of studies using electronic health record data is compelling, as this would enable large-scale cost-effective studies. However, based on previous studies, we anticipate that there would be partial correspondence between the peptide and full-length protein assays. This is expected as the full-length protein

recapitulates a larger antigen space than the peptides. While this does not invalidate any of the assays, they would be difficult to compare as they are designed to measure different affinity sets of antibodies. Still, should other groups be interested in attempting a record-based study, they could use the peptide sequences provided by us (e.g., in Fig 5b, Table S3, and Table 1) to set up the appropriate assay and evaluate its correspondence with the clinical assays of interest.

5. Did the authors do any other homology comparisons between other SARS-CoV2 proteins and their newly-identified autoantigens? Nucleocapsid in particular has been shown to be a molecular mimic in MIS-C (medRxiv 2023.05.26.23290373).

Authors' reply: We performed early comparisons with Nucleocapsid and did not find any notable sequence similarities.

6. Did any of the patients who developed new-onset aquaporin-4 autoantibodies develop CNS disease?

Authors' reply: No participant has reported new-onset CNS disease. We have indicated this in the updated demographic table.

7. The authors should consider also noting new-onset autoantibodies have been observed in patients with acute influenza, and association with anti-interferons and other cytokines (Feng, 36752204).

Authors' reply: We agree that this is a useful reference and have added it to the manuscript.

8. The vaccine data are interesting and important; however, it may be prudent to carefully characterize the interpretation as not supporting induction of autoantibodies or autoimmunity by this or other vaccines. There exists a strong anti-vaccine group of people who could seize on this finding and use it to show potential harm from vaccination. The authors should reiterate these results do not support this view, and moreover that the candidate antigens have not been linked to autoimmunity, long COVID, or vaccination.

Authors' reply: We fully agree with this assessment and highly appreciate this advice. We have added sentences to clarify this in the paragraph on SNURF in the Discussion, and have adjusted our wording throughout to preclude any out-of-context citation.

9. The authors should also supplement reference 15 by adding references to at least 2 papers showing absence of new or increased autoantibodies in vaccine trials (Arunachalam, Nature, PMID 34252919; and Sarin, JCI Insight, PMID 38456511). General absence of new autoantibodies in response to vaccines was also observed in a large cohort of patients with SLE and other autoimmune diseases (in press in JCI Insight).

Authors' reply: We have added these references to the above mentioned statements.

10. In the discussion, which is very well-written and balanced, the authors speculate regarding molecular mimicry. This is very reasonable and is supported by their findings. However, this reviewer recommends softening the language. For example instead of saying "indicative of molecular mimicry," they could say suggestive of, or consistent with a mechanism invoking molecular mimicry. This relates back to the vaccine link since the epitope is found both in the vaccine and the natural virus. The authors could also consider instead describing their findings

as "cross-reactive" epitopes since target antigens are intracellular and it is unclear how this would drive a break in tolerance. This is somewhat semantics but nevertheless worth considering.

Authors' reply: Although we agree that this is somewhat semantics, we have softened the language regarding molecular mimicry.

11. Several very large epidemiology studies have emerged showing an increased incidence of autoimmunity, including thyroid autoimmunity. The authors should consider linking their findings to these large epi studies. Examples include Tesch (PMID 37335408), Lim (PMID 37801317) and others.

Authors' reply: We agree that these are important links and have added these references to the Discussion.

REVIEWER COMMENTS

Reviewer #1 (Remarks to the Author):

The authors properly responded to all raised comments.

Authors' reply: We thank Reviewer #1 for the valuable feedback throughout the peer review process.

Reviewer #3 (Remarks to the Author):

I co-reviewed this manuscript with one of the reviewers who provided the listed reports. This is part of the Nature Communications initiative to facilitate training in peer review and to provide appropriate recognition for Early Career Researchers who co-review manuscripts.

Authors' reply: We thank Reviewer #3 for their participation.

Reviewer #4 (Remarks to the Author):

I have reviewed the revised manuscript as well as the critiques and rebuttal to all other reviewers. My initial review was very positive and most of my concerns have been addressed. The authors should look at some of the references that were suggested to be added since (I think) several that had been in MedRxiv are now published in peer-reviewed journals (eg Bodansky paper in Nature, the lupus vaccine paper in JCI Insight.

Authors' reply:

We thank Reviewer #4 for raising this point and have added the Bodansky Nature reference. Although we have looked at and incorporated the other suggested references that were published at the time of our previous response, we recognize that we did not sufficiently clearly tie our work to them, e.g., using the direct comparisons of response percentages suggested. To remedy this, **we have added a paragraph on study inter-study comparison to the Discussion**.

A concern remains regarding validation using whole antigens. It would not be at all difficult to pick a few key antigens that are clinically relevant such as thyroperoxidase and aquaporin-4 and validate the autoantibody results on an orthogonal platform such as ELISA, bead-based or other methods. Antigens are readily available and this group has pioneered micro-bead based AAb profiling. Two outcomes are possible, they either correlate or they don't. If they do, then other groups can validate on other cohorts. If not, then they can describe why there is a discrepancy.

Authors' reply:

While we greatly appreciate critique of our work, we were surprised to see this particular point raised once more, especially as Reviewer #4 previously stated:

> Reviewer #4:
> "This reviewer recognizes that the assays used here are research-grade, so a comprehensive validation effort is not indicated here."

As we detail below, we have addressed the questions regarding validation experiments in our previous revision, and we feel that Reviewer #4 has not thoroughly considered the issue. However, we see that we need to make our arguments clearer both in the peer review process and in the Discussion section of the manuscript.

Reviewer #4 has requested validation of our results using full-length protein antigens. This was indeed considered in our research process, but **after careful consideration we decided to first validate our results by the more specific method of peptide-based epitope mapping**. We have explained our reasoning in our previous point-by-point response to Reviewer #4 and feel that our reply has not been given due consideration. It is reproduced below for reference. Our main argument is that **full-length antigens encode a larger epitope space which confounds validation**. Furthermore, they are available in a multitude of formulations and variants, further contributing to variability of their epitope space. In addition, we are publishing the peptide sequences in Supplementary Table 4 to increase reproducibility, and we have added the sequences of the 22 protein fragments in this revision. These peptides and protein

fragments are easily ordered from protein synthesis services or can be shared by us on request, respectively. We have added this information in this revision.

> From our previous response to Reviewer #4:
> “However, based on previous studies, we anticipate that there would be partial correspondence between the peptide and full-length protein assays. This is expected as the full-length protein recapitulates a larger antigen space than the peptides**. While this does not invalidate any of the assays, they would be difficult to compare as they are designed to measure different affinity sets of antibodies.** Still, should other groups be interested in attempting a record-based study, they could use the peptide sequences provided by us (e.g., in Fig 5b, Table S3, and Table 1) to set up the appropriate assay and evaluate its correspondence with the clinical assays of interest.”

Although we stand by our argument that we have properly validated the detected antibodies within the present study using peptide antigens, we would like to clarify that we do not deny the potential value of analysis on full-length proteins. To this end, we have initiated a large international collaborative project in a large cohort study of patients with severe post-COVID-19 condition. In this research collaboration, we will use the peptides representing the herein validated epitopes to **enrich the autoantibodies of interest** using an in-house established procedure. These epitope enriched autoantibodies of interest will be thoroughly analyzed using full-length protein and clinical assays in parallel with unpurified sera for clinically oriented validation, which may enable clinical translation. **This is a major undertaking which cannot be performed in the presently studied cohorts due to limitations in availability of sample volume.** However, the international cohort will supply the required sample volumes, and will allow for further validation of these new-onset autoantibodies in severe post-COVID-19 syndrome. **This new project will take considerable time, likely several months.** In the light of recent publications on autoimmune conditions and autoantibodies in the post-COVID-19 condition, it is of considerable interest to the research community that the present results are published in a timely manner.

Notably, the main autoantibodies of interest in the present study, and consequently those most suited for further research, are those indicated in Figure 5, i.e., anti-CCDC63, NPC1, SNURF, TPO, and TRIM63 IgG. In particular, anti-AQP4 IgG, which is explicitly mentioned in the response by Reviewer #4, is not in this set. While anti-AQP4 IgG is indeed a highly clinically interesting autoantibody, it is a minor finding in the present study, and **we feel that the focus on AQP4 due to clinical relevance elsewhere is misdirected**. In contrast, as we do have evidence for the relevance of anti-TPO IgG in our study, we will make thorough comparisons of clinical and research-grade assays in our upcoming study.

Given the response from Reviewer #4, we recognize that we apparently have not articulated our reasoning regarding validation with sufficient clarity. To remedy this, **we have added a clarifying paragraph to the Discussion**.

Reviewer #4 (Remarks on code availability):

N/A
